# *APOE*, Aβ42, and tau differentially impact cognitive decline in Sporadic, *GBA1* and *LRRK2* Parkinson’s disease

**DOI:** 10.1038/s41531-026-01290-2

**Published:** 2026-02-23

**Authors:** Ragasudha Botta, Joseph J. Locascio, Rong Ye, Anna E. Goodheart, Stephen N. Gomperts

**Affiliations:** 1 Harvard Medical School, Boston, MA USA; 2 Department of Neurology, Massachusetts General Hospital, Boston, MA USA

**Keywords:** Diseases, Genetics, Neurology, Neuroscience

## Abstract

Cognitive impairment varies across sporadic Parkinson’s disease (PD) and the common genetic subtypes glucocerebrosidase (*GBA1*) and leucine-rich repeat kinase 2 (*LRRK2*) PD and is influenced by Apolipoprotein E (*APOE*) polymorphisms and Alzheimer’s disease (AD) co-pathology. However, the effects of *APOE* genotype, Aβ42 and tau on cognitive decline across these PD subtypes remain unclear. Using pooled longitudinal data across the PPMI and CPP cohorts, we examined the effects of *APOE* genotype and cerebrospinal fluid (CSF) Aβ42 and tau on cognitive decline across sporadic PD, *GBA1*-PD, *LRRK2*-PD, and healthy control (HC) subjects. Whereas in sporadic PD the *APOE ε4* allele was associated with faster cognitive decline than APOE *ε3* or *ε2* alleles, no *APOE* effect was observed in *GBA1*-PD or *LRRK2*-PD. While lower baseline CSF Aβ42 was linked to faster cognitive decline in all groups, higher baseline CSF pTau was associated with faster decline in sporadic PD and *LRRK2*-PD but not in *GBA1*-PD. These findings underscore differential vulnerabilities to *APOE* genotype and AD-related biomarkers among PD subtypes, a critical consideration for clinical trials targeting cognitive decline in PD.

## Introduction

Cognitive decline is common but not universal in Parkinson’s disease (PD), a genetically heterogeneous disease that can arise spontaneously as well as in association with mutations in PD risk genes including glucocerebrosidase (*GBA1*) and leucine rich repeat kinase 2 (*LRRK2*)^1–3^. Indeed, PD is associated with a 2.5-6 times higher odds of developing dementia compared to age-matched healthy controls^4^, with cumulative dementia prevalence rising from 17% within five years of diagnosis to 83% at 20 years^4^. PD genetic subtype has been found to be a strong contributor to cognitive decline, with risk of cognitive decline ~25% in sporadic PD, ~48% in *GBA1*-PD, and ~23% in *LRRK2*-PD^5–10^.

Apolipoprotein E (*APOE*) plays a key role in lipid metabolism, and *APOE* polymorphisms have been associated with AD risk in the general population, with the *APOE* ε4 genotype increasing AD risk three to four-fold, and the ε2 genotype reducing AD risk by half^11^. However, the effects of *APOE* polymorphisms on cognitive decline in sporadic PD remain inconclusive^12^, and their effects in *GBA1*-PD and *LRRK2*-PD are not well understood. Some evidence suggests that *APOE* ε4 accelerates cognitive decline in PD^13–16^, but this has been controversial^17,18^. In addition, the *APOE* ε2 allele has been linked to higher risk of PD dementia^19^, in contrast to its association with reduced dementia risk in the general population.

AD co-pathology is common in PD, with a prevalence of ~30–40%, and is a well-established risk factor for PD-associated cognitive decline^10,20–25^. When present, AD co-pathology reflected in low levels of CSF amyloid-beta 42 (Aβ42) and high levels of phosphorylated tau (pTau) (and thereby a higher pTau/Aβ42 ratio) has been associated with cognitive decline in prospective studies of PD blind to genotype^26–29^ (but see refs. ^30,31^). However, few studies focused on the impact of AD co-pathology on cognitive function have accounted for the influence of *GBA1* and *LRRK2* genotype, and findings have varied^32,33^.

To address these issues, we used the large PPMI and CPP datasets to investigate and compare the impact of *APOE* genotype and AD co-pathology reflected in CSF Aβ42 and pTau on cognitive course in sporadic PD, *GBA1*-PD, *LRRK2*-PD and healthy control (HC) participants.

## Results

### Demographic, clinical characteristics, and CSF biomarkers

**Fig. 1:**
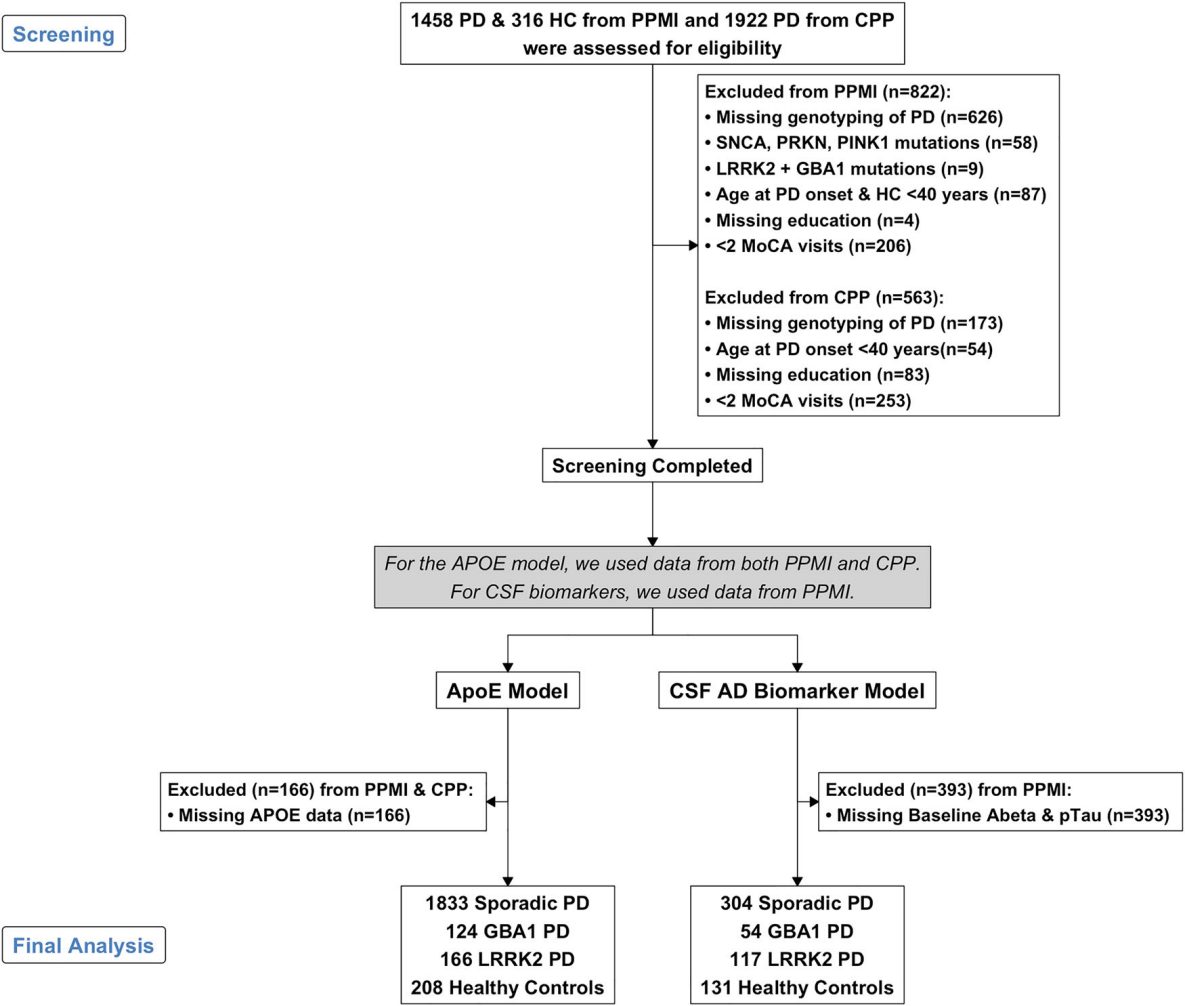
Study flow diagram. This flowchart illustrates the inclusion and exclusion process for the study. Boxes represent the number of individuals at each stage of the selection process, starting from the merged datasets and leading to the final cohorts used for each of the two primary analyses. Reasons for exclusion are provided at each step.

Baseline demographic and clinical characteristics of the 2,331 participants (Fig. 1) stratified by diagnostic group for *APOE* analyses are presented in Table 1. The baseline characteristics for each dataset are presented in Table S1. The mean age at baseline was 65.8 ± 8.9 years and varied significantly across the groups (ANOVA, p < 0.001). The proportion of males was 63.1% overall and varied across the groups (Chi square test, p < 0.01) as well, consistent with prior reports and lowest in the *LRRK2*-PD subgroup as shown previously^34^. The majority (97%) of participants were of White race.

**Table 1:** Baseline characteristics by diagnostic group

	Healthy Control (*N* = 208)	Sporadic PD (*N* = 1833)	*GBA1* PD (*N* = 124)	*LRRK2* PD (*N* = 166)	Total (*N* = 2331)	*P* value
Sex, n (%)						<0.01
Male	135 (64.9%)	1182 (64.5%)	74 (59.7%)	81 (48.8%)	1472 (63.1%)	
Female	73 (35.1%)	651 (35.5%)	50 (40.3%)	85 (51.2%)	859 (36.9%)	
Age, mean (SD) (Years)	63.0 (9.6)	66.5 (8.8)	63.4 (9.1)	64.5 (8.1)	65.8 (8.9)	<0.001
Education, n (%)						<0.001
12 years or less	29 (13.9%)	501 (27.3%)	21 (16.9%)	41 (24.7%)	592 (25.4%)	
More than 12 years of higher education	179 (86.1%)	1332 (72.7%)	103 (83.1%)	125 (75.3%)	1739 (74.6%)	
Age at Onset of PD (years)	NA	64.1 (8.8)	60.4 (9.4)	61.7 (8.3)	63.7 (8.8)	<0.001
Data missing	NA	40 (2.2%)	0 (0%)	0 (0%)	40 (1.7%)	
Duration of PD, mean (SD) (Years)	NA	2.4 (2.3)	2.9 (2.3)	2.8 (2.1)	2.4 (2.3)	<0.01
Data missing	NA	40 (2.2%)	0 (0%)	0 (0%)	40 (1.7%)	
MDS-UPDRS Part III Score, mean (SD)	1.3 (2.2)	21.6 (9.4)	24.9 (11.1)	21.1 (10.2)	17.5 (12.1)	<0.001
Data missing	0 (0%)	1268 (69.2%)	44 (35.5%)	40 (24.1%)	1352 (58.0%)	
*APOE*4 Status, n (%)						0.98
ε4 non-carriers	158 (76.0%)	1387 (75.7%)	97 (78.2%)	127 (76.5%)	1769 (75.9%)	
ε4 carriers	50 (24.0%)	446 (24.3%)	27 (21.8%)	39 (23.5%)	562 (24.1%)	
*APOE* Genotype, n (%)						0.10
*APOE* ε2	25 (12.0%)	253 (13.8%)	17 (13.7%)	23 (13.9%)	318 (13.6%)	
*APOE* ε3	133 (63.9%)	1134 (61.9%)	80 (64.5%)	104 (62.7%)	1451 (62.2%)	
*APOE* ε4	50 (24.0%)	446 (24.3%)	27 (21.8%)	39 (23.5%)	562 (24.1%)	
Montreal Cognitive Assessment Score, mean (SD)	28.0 (1.5)	25.7 (3.2)	25.8 (2.9)	25.9 (3.3)	25.9 (3.1)	<0.001

Abbreviations: *PD* Parkinson’s Disease, *APOE* Apolipoprotein E, *MDS_UPDRS* MDS-Unified Parkinson’s Disease rating scale, *GBA1* Glucocerebrosidase mutation, *LRRK2* Leucine-rich repeat kinase 2, *SD* standard deviation.

Results are presented as number (%) or mean (SD). Percentages may not sum to 100 due to rounding. *APOE* genotypes are categorized as follows: *APOE* ε3 (two copies of the ε3 allele), *APOE* ε2 (ε2/ε2 or ε2/ε3), and *APOE* ε4 (ε3/ε4, ε2/ε4, or ε4/ε4).

The mean follow-up period was 3.6 ± 3.3 years. Among PD subgroups, the *GBA1*-PD group exhibited the youngest disease onset (60.4 ± 9.4 years, *p* < 0.001) and the longest disease duration (2.9 ± 2.3 years, *p* < 0.01). Across PD subgroups, baseline MoCA scores were consistently lower than in HC (sporadic PD 25.7 ± 3.2, *GBA1*-PD 25.8 ± 2.9, *LRRK2*-PD 25.9 ± 3.3, HC 28.0 ± 1.5, *p* < 0.001). Baseline MDS-UPDRS Part III scores were comparable across PD subgroups and higher than HC (sporadic PD 21.6 ± 9.4, *GBA1*-PD 24.9 ± 11.1, *LRRK2*-PD 21.1 ± 10.2, HC 1.3 ± 2.2, *p* < 0.001). Baseline MoCA scores were comparable across *APOE* genotypes of the PD subgroups (Table S2).

The baseline characteristics of participants with CSF AD biomarkers are shown in Tables S3 and S4. HC exhibited the highest levels of CSF Aβ42 (887 ± 264 pg/ml), while *GBA1*-PD had the lowest levels (739 ± 270 pg/ml, *p* < 0.01). Aβ42 levels significantly differed across *APOE* genotypes (*p* < 0.001), lowest in *APOE* ε4 carriers and highest in *APOE* ε3 carriers. pTau levels in the PD groups were significantly lower than in HC (p < 0.001) and were unaffected by *APOE* genotype.

### Predictive effects of APOE polymorphism and diagnostic group on cognitive decline

**Fig. 2:**
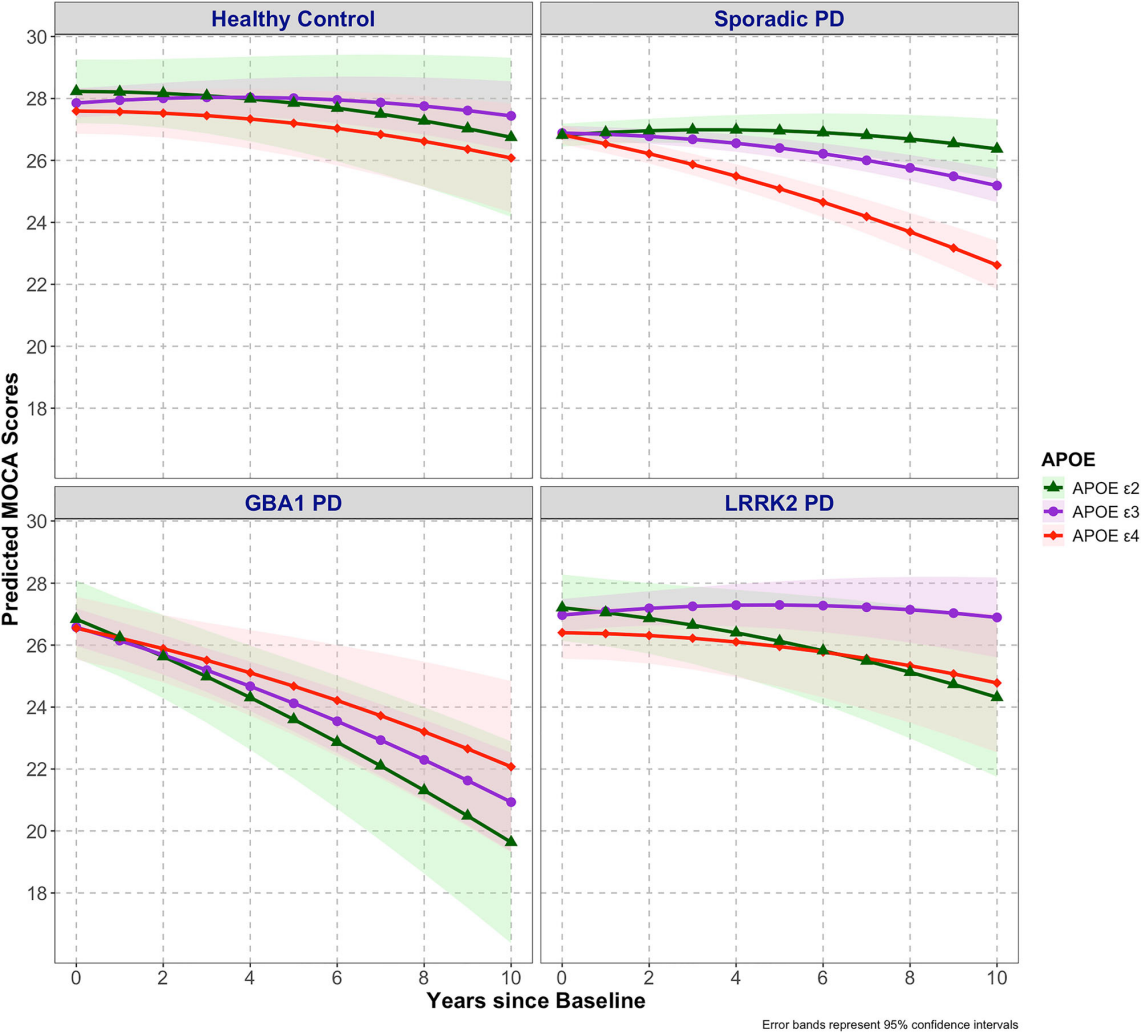
Effect of APOE polymorphism on cognitive decline in PD subgroups and healthy controls. The effects of *APOE* polymorphism on MoCA score trajectories are shown across Parkinson’s disease (PD) subgroups and healthy controls (HC). In sporadic PD, *APOE*4 genotype was associated with significantly faster decline in MoCA scores than *APOE*3 and *APOE*2 groups. *APOE* genotype was not associated with cognitive decline in the HC, *GBA1* PD, or *LRRK2* PD groups. Values graphed are those predicted by model fixed effects holding constant covariates of baseline age, sex, education and duration of PD at baseline. Data shown reflect results for mean age (64.2 years), male sex, education >12 years and mean PD duration at baseline (1.7 years). Colored bands indicate 95% confidence intervals. Sample sizes included in the model were: Healthy Control, *n* = 208 Sporadic PD, *n* = 1883; ***GBA1***-PD, *n* = 124; and ***LRRK2***-PD, *n* = 166.

*APOE* model: We first examined the effects of *APOE* genotype, diagnosis, time, covariates, and interaction effects on longitudinal cognitive trajectories (as assessed by the Montreal Cognitive Assessment; MoCA). Missingness in baseline covariates was minimal and was greatest for education (missing in 3.5% of participants) and disease duration (missing in ~1.7%). To address missing data, we conducted a complete case analysis, including only participants with non-missing values for education and disease duration in the model. After eliminating non-significant higher-order terms, significant main effects were observed for baseline age (F (1,2158.97) = 206.29, *p* < 0.0001), education (F (1,2205.80) = 131.23, *p* < 0.0001), sex (F (1, 2211.48) = 40.18, *p* < 0.0001) and duration of PD (F (1,2296.44) = 13.95, *p* < 0.0001). Individuals with ≤ 12 years of education scored 1.55 MoCA points lower than those with > 12 years (*p* < 0.001) overall (but note interaction below). Males had lower baseline MoCA scores than females. Those with longer disease duration at baseline had lower baseline MoCA scores. Older participants had lower mean MoCA overall, but the interaction between age and linear time was also strong (F (1, 1140.68) = 168.89, *p* < 0.001), such that older age at baseline was associated with faster decline on MoCA scores (Fig. S1). A significant effect of quadratic time was also found (F (1, 8824.26) = 67.22, *p* < 0.001) reflecting a general tendency for rate of decline to accelerate slightly over time (Fig. 2). Another significant fixed effect was the two-way interaction between education and linear time (F (1, 1176.60) = 8.66, *p* = 0.03), such that higher education was associated with a slower decline on MoCA.

A significant three-way interaction was observed between diagnosis, *APOE* genotype, and time (F (6867.12) = 3.49) *p* = 0.002; Fig. 2, Table S5) in the mixed-effects model, highlighting the differential impact of *APOE* genotypes on cognitive trajectories across the diagnostic subgroups. As Fig. 2 illustrates, the 3-way interaction was reflected by more fanning out of MoCA scores for sporadic PD and *LRRK2*-PD groups across *APOE* genotypes and time, as compared to the other groups. Table 2 presents the partial unstandardized regression coefficients, while type III corresponding ANOVA F-test results are shown in Table S5.

**Table 2:** Unstandardized & standardized partial regression coefficients for the fixed effects on cognitive decline (MoCA) over time in the APOE genotype model

	Unstandardized partial regression coefficients			Standardized partial regression coefficients		
Factors	**Estimates**	**95% CI**	**p**	**Estimates**	**95% CI**	**p**
(Intercept)	32.40	31.45 – 33.35	<0.001	26.22	25.63 – 26.82	<0.001
Reference: Diagnosis [Healthy Control]						
Diagnosis [*GBA1*]	-1.27	-2.02 – -0.53	0.001	-2.83	-3.71 – -1.96	<0.001
Diagnosis [*LRRK2*]	-0.89	-1.56 – -0.21	0.010	-0.78	-1.58 – 0.01	0.05
Diagnosis [Sporadic PD]	-0.97	-1.45 – -0.49	<0.001	-1.35	-1.92 – -0.79	<0.001
Time	1.39	1.14 – 1.64	<0.001	-0.22	-0.53 – 0.08	0.146
Time^2	-0.01	-0.02 – -0.01	<0.001	-0.10	-0.14 – -0.06	<0.001
Reference: *APOE* [*APOE* ε3]						
*APOE* [*APOE* ε2]	0.37	-0.73 – 1.47	0.51	0.06	-1.25 – 1.36	0.93
*APOE* [*APOE* ε4]	-0.26	-1.09 – 0.57	0.54	-0.59	-1.57 – 0.40	0.24
Age at Baseline	-0.09	-0.10 – -0.08	<0.001	-1.38	-1.51 – -1.25	<0.001
Reference: Education [Less than 12 years]						
Education [More than 12 years or higher education]	1.50	1.24 – 1.76	<0.001	1.81	1.51 – 2.12	<0.001
Reference: Sex [Female]						
Sex [Male]	-0.73	-0.96 – -0.50	<0.001	-0.73	-0.96 – -0.50	<0.001
Duration of PD at Baseline	-0.10	-0.15 – -0.05	<0.001	-0.20	-0.30 – -0.09	<0.001
2-way Interactions:						
Diagnosis [*GBA1*] × Time	-0.52	-0.71 – -0.34	<0.001	-1.35	-1.83 – -0.88	<0.001
Diagnosis [*LRRK2*] × Time	0.03	-0.12 – 0.19	0.67	0.09	-0.32 – 0.49	0.67
Diagnosis [Sporadic PD] ×Time	-0.13	-0.24 – -0.02	0.02	-0.33	-0.62 – -0.04	0.02
Diagnosis [*GBA1*] × *APOE* [*APOE* ε2]	-0.12	-1.88 – 1.64	0.90	-0.26	-2.35 – 1.82	0.81
Diagnosis [*LRRK2*] × *APOE* [*APOE* ε2]	-0.14	-1.74 – 1.47	0.87	-0.66	-2.55 – 1.23	0.50
Diagnosis [Sporadic PD] ×*APOE* [*APOE* ε2]	-0.44	-1.60 – 0.72	0.46	0.25	-1.13 – 1.62	0.72
Diagnosis [*GBA1*] × *APOE* [*APOE* ε4]	0.23	-1.20 – 1.65	0.76	0.90	-0.79 – 2.60	0.30
Diagnosis [*LRRK2*] × *APOE* [*APOE* ε4]	-0.31	-1.58 – 0.97	0.64	-0.44	-1.96 – 1.08	0.57
Diagnosis [Sporadic PD] ×*APOE* [*APOE* ε4]	0.20	-0.68 – 1.09	0.66	-0.22	-1.27 – 0.83	0.68
Time × *APOE* [*APOE* ε2]	-0.11	-0.37 – 0.16	0.43	-0.28	-0.95 – 0.40	0.43
Time × *APOE* [*APOE* ε4]	-0.11	-0.30 – 0.08	0.26	-0.28	-0.78 – 0.21	0.26
Time × Age at Baseline	-0.02	-0.03 – -0.02	<0.001	-0.50	-0.57 – -0.42	<0.001
Time × Education [More than12 years or higher education]	0.10	0.03 – 0.17	<0.001	0.27	0.09 – 0.45	<0.001
3-way Interactions:						
(Diagnosis [*GBA1*] × Time) × *APOE* [*APOE* ε2]	-0.05	-0.48 – 0.39	0.83	-0.13	-1.25 – 1.00	0.83
(Diagnosis *[LRRK2*] × Time) × *APOE* [*APOE* ε2]	-0.18	-0.55 – 0.20	0.36	-0.45	-1.42 – 0.52	0.36
(Diagnosis [Sporadic PD] × Time) × *APOE* [*APOE* ε2]	0.23	-0.05 – 0.51	0.11	0.60	-0.12 – 1.32	0.11
(Diagnosis [*GBA1*] × Time) × *APOE* [*APOE* ε4]	0.23	-0.14 – 0.59	0.22	0.59	-0.35 – 1.53	0.22
(Diagnosis *[LRRK2*] × Time) × *APOE* [*APOE* ε4]	-0.04	-0.36 – 0.27	0.78	-0.12	-0.92 – 0.69	0.78
(Diagnosis [Sporadic PD] ×Time) × *APOE* [*APOE* ε4]	-0.14	-0.35 – 0.07	0.18	-0.36	-0.90 – 0.17	0.18

Abbreviations: *APOE* Apolipoprotein E, *GBA1* Glucocerebrosidase mutation, *LRRK2* Leucine-rich repeat kinase 2, Time^2 is quadratic time.

Post hoc pairwise contrasts using Tukey adjustment for multiple comparisons identified significant differences in cognitive trajectories between *APOE* genotypes within the sporadic PD subgroup only (Table S6). *APOE* ε4 carriers in sporadic PD declined faster than ε3 carriers (estimated difference in linear terms = -0.43, SE = 0.15, *p* = 0.01), with a significant difference first evident at 1.49 years. Differences between *APOE* ε4 and ε2 carriers were also significant at this time point with *APOE* ε2 carriers demonstrating higher MoCA scores than ε4 carriers (estimated difference in linear terms = 0.55, SE = 0.22, *p* = 0.03) and persisted (*p* < 0.05). No significant differences were observed between *APOE* ε3 and ε2 carriers at any time point (*p* > 0.05). No significant *APOE* genotype effects were found in HC, *GBA1*-PD, or *LRRK2*-PD. Fixed effects explained 22.2% of the variance in MoCA in this *APOE* model, indicating that a substantial portion of cognitive variability was attributable to measurable clinical and genetic predictors.

Similar results were observed when we examined the predictive effect of *APOE* ε4 carrier status versus non-*APOE* ε4 (i.e., *APOE* ε3 and ε2 pooled) as a binary variable on cognitive impairment across the groups (Table S7, Fig. S2). The three-way interaction between diagnosis, *APOE* ε4 status, and time was significant in the mixed-effects model (F (3887.12) = 3.37, *p* = 0.02). *APOE* ε4 carriers in the sporadic PD subgroup alone exhibited greater cognitive decline compared to non-carriers (Table S8). Partial unstandardized regression coefficients are available in Table S9.

To account for cohort-level variability, we re-ran the *APOE* model including cohort as a fixed effect. While cohort was a significant predictor of MoCA scores at baseline (F (1, 2217.01) = 43.25, *p* < 0.001), its inclusion did not alter the overall pattern of results. We investigated the predictive effect of *APOE* ε4 carrier status compared to non-carriers. The interaction between diagnosis, time, and *APOE* ε4 remained significant (F (3900.90) = 3.29, *p* = 0.02). Notably, disease duration did not retain significance in this adjusted model and was excluded. The explained variance was nearly unchanged, with a marginal R² of 22.6% compared to 22.2% in the original model, and the predicted trajectories were visually similar (Table S10 & Fig. S3).

Model-derived contrasts quantifying the difference in annual MoCA decline (points/year) associated with APOE ε4 carriage (ε4 vs non-carrier) within each PD subgroup are shown in Table S11.

Together, these results show that the effects of *APOE* genotype on cognitive course are robust in sporadic PD but not in *GBA1*-PD, *LRRK2*-PD or HC.

### Effects of CSF Aβ42 and pTau and Diagnostic Group on Cognitive Decline

**Fig. 3:**
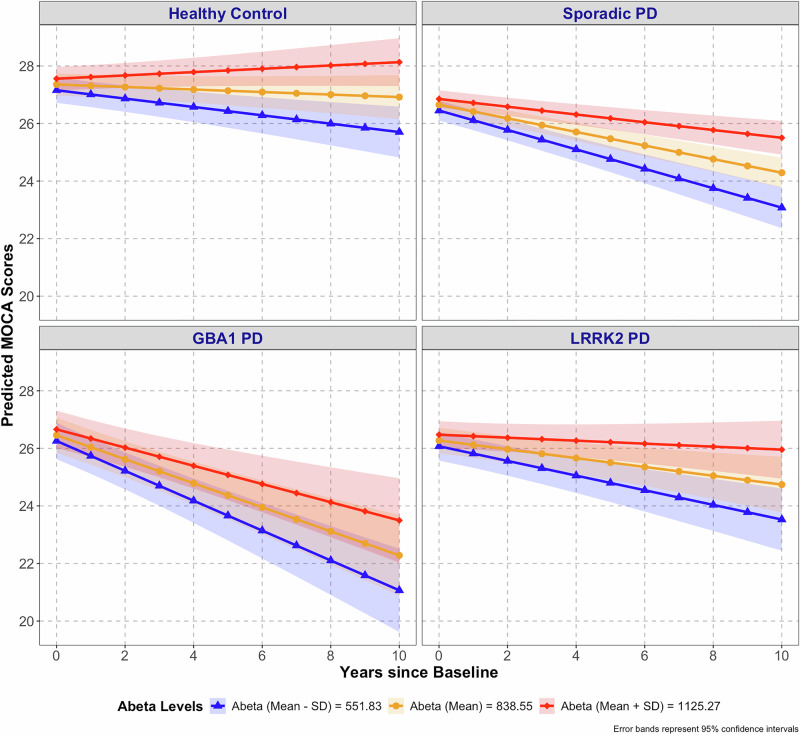
Effect of baseline Aβ42 levels on cognitive decline in PD subgroups and healthy controls. The impact of baseline Aβ42 levels (Mean ± SD) on MoCA score trajectories is shown across Parkinson’s disease (PD) subgroups and healthy controls (HC) for three hypothetical groups based on Aβ42 levels: mean, mean + one SD, and mean - one SD. Lower Aβ42 levels were associated with greater cognitive decline in all groups. HC and LRRK2-PD cases with the highest Aβ42 levels had the greatest cognitive resilience, while GBA1-PD with the lowest Aβ42 levels had the most pronounced cognitive decline. Values graphed are those predicted by model fixed effects holding constant covariates of baseline age, sex, education, duration of PD and baseline pTau. Data shown reflect results for mean age (62.2 years), male sex, mean education (15.7 years), mean duration of PD at baseline (1.4 years), and mean pTau (15.3 pg/ml). Colored bands indicate 95% confidence intervals. Sample sizes included in the model were: Healthy Control, *n* = 131; Sporadic PD, *n* = 304; *GBA1*-PD, *n* = 54; and *LRRK2*-PD, *n* = 117.

CSF AD Biomarker Model: We next examined the effects of CSF biomarkers of AD co-pathology (baseline Aβ42, pTau), diagnosis (HC, sporadic PD, *GBA1*-PD, and *LRRK2*-PD), time, covariates (baseline age, education, sex, and duration of PD at baseline), and interaction effects on longitudinal cognitive trajectories reflected by changes in MoCA score over time. After eliminating non-significant higher-order terms, a significant main effect was observed for sex (F (1567.34) = 6.45, *p* = 0.01) with males exhibiting greater cognitive decline over time compared to females (Table S12). Additionally, significant two-way interactions were found, including baseline age and time (F (1322.19) = 39.44, *p* < 0.0001) with older declining faster, years of education and time (F (1352.44) = 6.76, *p* = 0.01) with less educated declining faster, and duration of PD and time (F (1393.16) = 8.41, *p* < 0.01). Longer disease duration was associated with a slower decline in MoCA scores. This effect was driven by a small number of subjects with the longest disease durations. A significant interaction between time and Aβ42 levels (F (1335.25) = 30.58, *p* < 0.0001) was also observed, such that lower Aβ42 levels were associated with faster cognitive decline across diagnostic groups. Lower baseline levels of Aβ42 (Mean - SD), 551.83 pg/ml) were associated with significant reduction in MoCA scores compared to higher levels ((Mean + SD), 1125.27 pg/ml) as early as 0.39 years from baseline and persisted (Fig. 3). No significant three-way interaction was detected between diagnosis, baseline Aβ42, and time.

When Aβ42 was dichotomized using PD-derived Aβ42 cut-offs (see Methods), low Aβ42 was consistently associated with significantly worse cognitive trajectories across all diagnostic groups (Tables S13-S15; Figure S4).

**Fig. 4:**
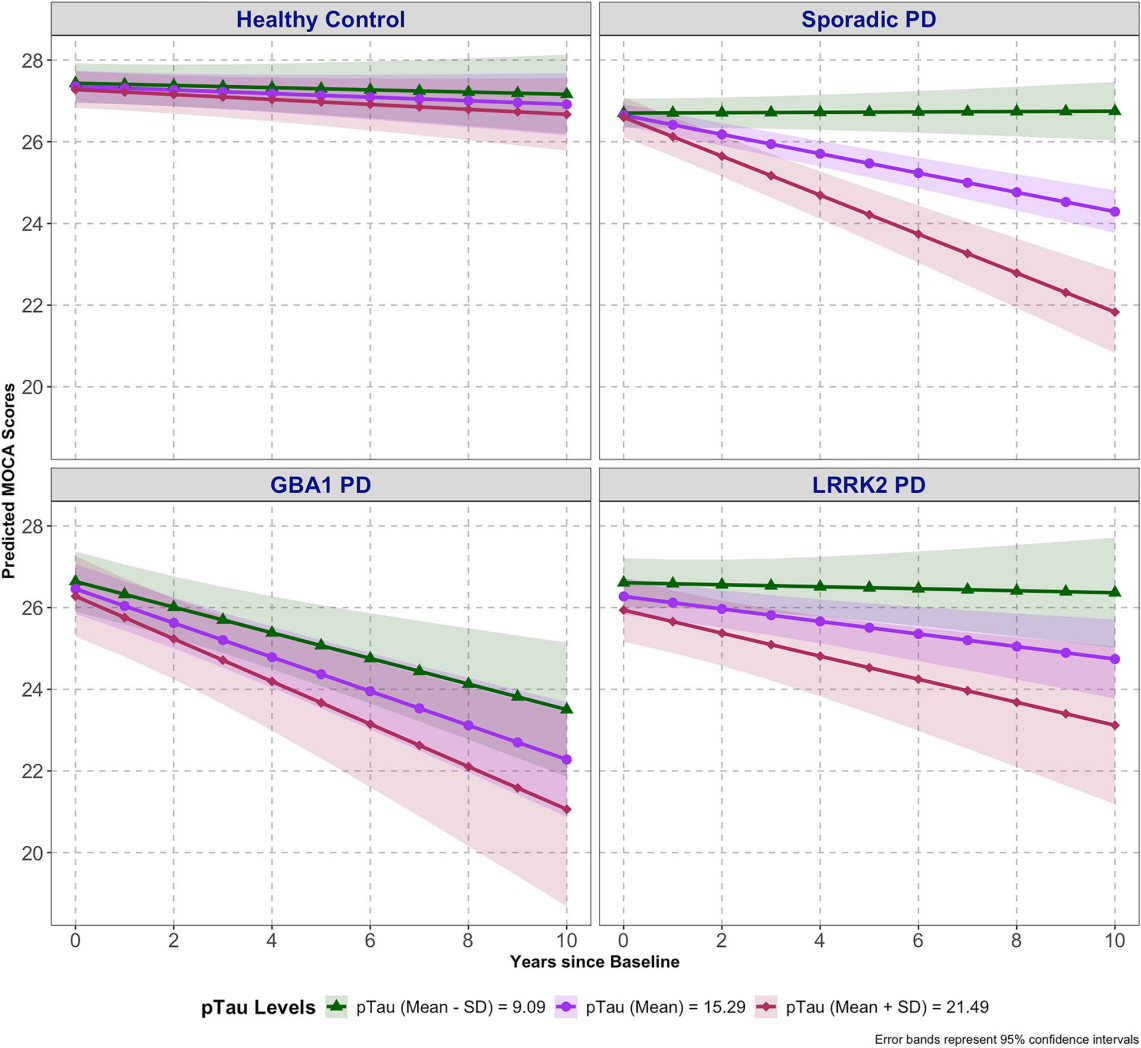
Effect of baseline pTau levels on cognitive decline in PD subgroups and healthy controls. The effect of baseline pTau levels (Mean ± SD) on MoCA score trajectories is shown across Parkinson’s disease (PD) subgroups and healthy controls (HC) for three hypothetical groups based on pTau levels: mean, mean + one SD, and mean - one SD. Higher baseline pTau levels were associated with faster cognitive decline in sporadic PD and *LRRK2*-PD, and this effect was greatest for sporadic PD. Baseline pTau was not significantly associated with rate of cognitive decline in *GBA1* PD or HC. Values graphed are those predicted by model fixed effects holding constant covariates of baseline age, sex, education, duration of PD and baseline Aβ42. Data shown reflect results for mean age (62.2 years), male sex, mean education (15.7 years), mean duration of PD at baseline (1.4 years) and mean Aβ42 (838.6 pg/ml). Colored bands indicate 95% confidence intervals. Sample sizes included in the model were: Healthy Control, *n* = 131; Sporadic PD, *n* = 304; *GBA1*-PD, *n* = 54; and *LRRK2*-PD, *n* = 117.

A significant three-way interaction was observed between diagnosis, baseline pTau levels, and time (F (3537.18) = 10.24, *p* < 0.0001) (Table S12; Fig. 4). In post hoc analyses, higher pTau levels in sporadic PD and in *LRRK2*-PD were significantly associated with faster cognitive decline. MoCA values between sporadic PD subjects with 1 SD higher (mean + SD) vs. 1 SD lower (mean - SD) CSF pTau significantly diverged by year 2 (estimated difference in linear slope = 1.07, SE = 0.35, *p* < 0.01). In the *LRRK2*-PD subgroup, significant differences emerged by year 4 (estimated difference in linear slope = 0.70, SE = 0.28, *p* = 0.04). In contrast, no significant association was found between pTau levels and cognitive decline in *GBA1*-PD or HC. Partial unstandardized and standardized regression coefficients are provided in Table S16. Fixed effects explained 22.8% of the variance in MoCA in this CSF AD biomarker model.

In contrast to these PD subgroup-specific effects of CSF pTau in continuous analyses, when pTau was dichotomized using PD-derived pTau cut-offs, higher pTau levels were significantly associated with faster cognitive decline in all PD subtypes (Table S17; Fig. S5).

Model-derived contrasts quantifying the difference in annual MoCA decline (points/year) associated with lower Aβ42 (Mean - SD vs Mean) and higher pTau (Mean + SD vs Mean) within each PD subgroup are shown in Tables S18 and S19.

Together, these results show that the effect of baseline Aβ42 levels on cognitive decline is additive to the effect of PD subtype (sporadic PD, *GBA1*-PD, or *LRRK2*-PD), whereas the effect of pTau in continuous analyses varies by PD subtype and is strongest in sporadic PD, detectable but weaker in *LRRK2*-PD, and nonsignificant in *GBA1*-PD.

### CSF Aβ42 as a Mediator of APOE ε4-Related Cognitive Decline in sporadic PD

In sporadic PD, mediation analysis revealed a significant indirect pathway linking *APOE ε4* carrier status to faster cognitive decline through baseline CSF Aβ42 levels (average causal mediation effect [ACME] = –0.056, 95% CI –0.073 to –0.039, *p* < 0.001), accounting for approximately 33% of the total effect (See Statistical analysis section” below). The direct effect of *APOE ε4* on cognitive decline, independent of CSF Aβ42, was larger in magnitude (average direct effect [ADE] = –0.113, 95% CI –0.179 to –0.049, *p* < 0.01), representing the remaining ~67% of the total effect (total effect = –0.169, 95% CI –0.238 to –0.100, *p* < 0.001). These findings suggest that lower baseline CSF Aβ42 levels partially mediated the association between APOE *ε4* carrier status and faster cognitive decline in sporadic PD.

In an analogous analysis focused on evaluating CSF pTau as a mediator of the effect of *APOE ε4* carrier status on cognitive decline in sporadic PD, baseline pTau accounted for only a small and statistically non-significant proportion of the association between *APOE* ε4 status and longitudinal cognitive decline.

## Discussion

*APOE* genotype and AD co-pathology have each been identified as risk factors for cognitive decline in PD, but the distinct effects of these risk factors in sporadic PD, *GBA1*-PD, and *LRRK2*-PD have been unclear. Given the prevalence of *GBA1*-PD, and *LRRK2*-PD, which account for an estimated 3-10% of PD overall^35^, we studied this question in the large PPMI and CPP cohorts. Our findings demonstrate differential effects of *APOE* genotype, Aβ42, and pTau on cognitive outcome in these PD subtypes.

*APOE* genotype impacted cognitive course strongly and selectively within the sporadic PD group, where *APOE* ε4 was associated with a 3-point lower MoCA score over 10 years than ε3 and ε2. These results confirm and extend previous reports linking *APOE* ε4 carrier status to accelerated cognitive decline in sporadic PD^14,36,37^, beyond what is observed in the general population^38–40^. The present findings are also consistent with prior work in *GBA1*-PD, where *APOE* ε4 carrier status was not linked to cognitive decline^41^. This observation raises the possibility of an epistatic interaction between *APOE* and *GBA1* on cognitive outcome in PD. On the other hand, the present results contrast with those of a recent study reporting a combined influence of *APOE* ε4 and *GBA1* mutations on cognitive course^42^. As that study included *GBA1* variants of uncertain significance, and as some of those variants may not affect *GBA1* function, sporadic PD cases may have been inadvertently included. Further work is needed to resolve this question. The present findings also suggest that *LRRK2*-PD is spared from a strong detrimental effect of *APOE* ε4 on cognitive course.

To date, few studies have investigated the effect of *APOE* ε2 on cognitive decline across PD subtypes. Although *APOE* ε2 has been associated with reduced rates of cognitive decline in the general population^43,44^, we did not detect such an effect in any PD subtype. These results confirm and extend similar observations in sporadic PD^45^.

The large effects of *APOE* genotype on cognitive trajectory in sporadic PD that parallel and exceed the magnitude observed in HC suggest that accounting for *APOE* genotype in cognition-focused clinical trials of sporadic PD is likely to prove useful. Doing so may reduce the variance significantly, improving sensitivity to detect a therapeutic effect. PD studies focused on *APOE* are also likely to benefit from consideration of *GBA1* and *LRRK2* genotype, where *APOE* genotype appears to be less influential than in sporadic PD on cognitive outcomes.

AD co-pathology is common in PD, and amyloid burden reflected in low levels of CSF Aβ42 and elevated Pittsburgh compound-B (PiB) PET have been consistently associated with faster cognitive decline in PD^25,30^, but the effects of AD co-pathology in *GBA1*-PD and *LRRK2*-PD have been less clear. We found that the impact of Aβ42 levels on cognitive decline was similar across sporadic PD, *GBA1*-PD, *LRRK2*-PD, and HC, providing an additive effect to the cognitive trajectory of each PD subtype that translated to a 2-point difference in MoCA across 10 years. Across PD subtypes, this effect accentuated cognitive trajectories, which ranged from cognitive stability in *LRRK2*-PD carriers with high levels of Aβ42, to a 5-point MoCA score decline over 10 years in *GBA1*-PD with low levels of Aβ42.

Exploratory mediation analyses suggested that in sporadic PD, nearly one-third of the detrimental influence of *APOE ε4* on MoCA slope was attributable to its association with lower baseline Aβ42 levels, while the rest was explained by Aβ42-independent mechanisms. Although this observation should be interpreted cautiously, given the observational design and the smaller biomarker subset, it supports the possibility of partially overlapping *APOE* and Aβ42-related pathways in sporadic PD.

Consistent with prior studies that have shown lower pTau levels in PD compared to HC^46^, we observed lower pTau in PD as well (Table S3). While the mechanism is unclear, this pTau reduction mirrors a parallel reduction in total tau.

Previous research has linked elevated CSF pTau to cognitive decline in PD^32,47,48^, but its impact in *GBA1*-PD and *LRRK2*-PD has been less studied. Like *APOE* genotype, the effect of CSF pTau on cognitive decline also depended upon PD subtype: In continuous analyses, higher pTau was associated with a pronounced acceleration of cognitive decline in sporadic PD, with a smaller detrimental effect in *LRRK2*-PD as well. These results are consistent with neuropathological work showing that the severity of AD tau pathology in *LRRK2*-PD correlates with progression to dementia.^49^ In contrast, pTau levels did not appreciably impact cognitive decline in *GBA1*-PD.

In AD cohorts, CSF pTau cut-offs commonly used for biological stratification are substantially above 24 pg/mL, depending on assay and cohort, reflecting established AD-type tau pathology. As the CSF pTau levels were markedly lower in the PD subgroups (Table S3), the striking impact of CSF pTau on cognitive decline in sporadic PD suggests that even modest variation in pTau within a non-AD range may be clinically relevant for cognitive decline in sporadic PD.

Consistent with prior reports, age, sex, education, and duration of disease were significantly associated with cognitive trajectories in PD in these analyses, underscoring their importance. Young age, female sex and higher education offset some of the apparent pernicious effects of a worse PD subtype and/or a worse *APOE* genotype. Additionally, in a sensitivity analysis in which PD duration was omitted from the APOE and CSF models, the findings remained unchanged (data not shown).

This study has several strengths, including a large sample size, a longitudinal design, and an extended follow-up period, that facilitate evaluation of *APOE* genotype and CSF Aβ and pTau data on cognitive decline across PD subtypes. The consideration of both *LRRK2*-PD and *GBA1*-PD contrasted with sporadic PD and HC is an additional strength, as is the study of exclusively pathogenic variants of *LRRK2* and *GBA1*. In our models, fixed effects accounted for a sizeable proportion of the variance in longitudinal cognitive trajectories. However, there are also several limitations. We studied clinically diagnosed cases of PD, and although fibrillar alpha-synuclein is not consistently observed in *LRRK2*-PD, future work with fibrillar alpha-synuclein-confirmed sporadic PD will be of value. Despite the overall sample size, the ε2 and ε4 subgroups within the *GBA1*-PD and *LRRK2*-PD cohorts were relatively small. Although the ε2/ε4 genotype was categorized under ε4 carriers, potentially biasing results toward the null, sensitivity analyses including and excluding these cases demonstrated no significant impact on ε2- or ε4-associated findings. Due to lack of data in one of the cohorts, we were unable to adjust for medications that may affect cognition. Another potential limitation is the variable presence of missing data, although the mixed-effects models used here aimed to account for this variability. For longitudinal outcome data, some missingness was expected due to missed follow-up visits. To handle this, we employed Restricted Maximum Likelihood (REML) estimation within our linear mixed-effects models, which allows for the inclusion of all available data without requiring imputation, under the assumption that data are missing at random. This method leverages all observed outcome data per participant and provides less biased estimates than traditional complete case analysis. A further limitation is the minimal racial diversity in the cohort, which limits the generalizability of *APOE ε4*–related findings to other populations and prevents assessment of racial variation in genetic risk associations. Despite these limitations, we were able to detect robust effects of *APOE* genotype, CSF Aβ, and CSF pTau on cognitive trajectories in *GBA1*-PD, *LRRK2*-PD, and sporadic PD.

Together, our findings suggest that the effects of *APOE* genotype and AD-related CSF Aβ42 and tau on cognitive decline depend on PD subtype. In sporadic PD but not in *GBA1*-PD or *LRRK2*-PD, *APOE* ε4 was linked to faster cognitive decline. Whereas lower baseline levels of Aβ42 provided a fixed, additive effect to diagnosis on rate of cognitive decline across all groups, the effect of higher baseline pTau on cognitive decline was greatest in sporadic PD, intermediate in *LRRK2*-PD, and minimal in *GBA1*-PD. These observations warrant further investigation. Accounting for *APOE* ε4, Aβ42, and tau along with PD genetic subtype in clinical trials targeting PD-associated cognitive impairment has potential to reduce variance and improve the ability to detect a therapeutic effect.

## Methods

### Study Design & Setting

We employed a retrospective longitudinal cohort design to investigate the relationship between PD subtype, *APOE* genotype, CSF Aβ42 and tau biomarkers, and cognitive trajectories in patients with PD compared to healthy individuals. We pooled data from the Parkinson’s Progression Markers Initiative (PPMI, NCT01141023) and Tracking Parkinson’s/PRoBAND (NCT02881099), part of the Integrated Parkinson’s Database of Critical Path for Parkinson’s (CPP)^50^. From the PPMI cohort (50 centers), we included de novo sporadic PD and both drug-naïve and treated genetic PD patients, diagnosed within two years and classified as Hoehn and Yahr stages 1–2. Participants were followed for up to 10 years, with data analyzed from 2010 to the October 2024 data freeze. The CPP cohort (72 sites) included drug-naïve and treated PD patients diagnosed within 3.5 years, with follow-up extending up to 7.5 years from February 2012 to the 2022 data freeze. The PPMI and CPP studies obtained approval from the institutional review boards at their participating sites and were conducted in accordance with the Declaration of Helsinki. All participants provided signed informed consent.

### Participants

We screened 1,458 PD subjects and 316 HC from the PPMI and 1,922 PD subjects from the CPP. Inclusion criteria included a diagnosis of sporadic PD, *GBA1*-PD, *LRRK2*-PD, or HC, age 40 years or older, and at least two study visits with Montreal Cognitive Assessment (MoCA) testing spaced at least one year apart. In the *GBA1*-PD and *LRRK2*-PD groups, only those with pathogenic variants were included. HC had no neurological deficits and no first-degree relatives diagnosed with PD. 822 participants were excluded from the PPMI and 563 from the CPP based on these predefined criteria. The final analysis included 1833 sporadic PD, 124 *GBA1*-PD, 166 *LRRK2*-PD, and 208 HC participants. The study flow chart is shown in Fig. 1. The proportions of MoCA assessment visits across the PD subgroups are shown in Table S20. Spaghetti plots showing individual trajectories and variability in visit frequency across diagnostic groups are included in the Supplement (Figure S6).

### Procedures and Measures (Clinical assessments, APOE genotyping, and CSF biomarker measurement)

The primary outcome was cognitive function, assessed using the Montreal Cognitive Assessment (MoCA), a 30-point screening tool evaluating multiple cognitive domains, including memory, executive function, visuospatial skills, and language (higher scores indicate better function)^51^. MoCA was acquired annually for PPMI participants and semi-annually for CPP participants. In the PPMI cohort, we treated time as a continuous variable using decimal values (e.g., 1.53, 2.70 years), based on each participant’s actual time from baseline at each visit. For the CPP dataset, time was only available in discrete 6-month intervals (e.g., 0.5, 2 years). The primary exposure variables were *APOE* genotype and CSF biomarkers (Aβ42, pTau). Covariates included age, sex, education, and duration of PD^52^^,53^.

The key pathogenic *GBA1* variants included in the study were p.R535H (rs80356773), p.R502C (rs80356771), p.L483P (rs421016), p.N409S (rs76763715), IVS2 + 1 G > A (rs104886460), and p.L29Afs 18 (rs387906315). These variants show elevated odds ratios ranging from 3.58 to 10.49, indicating a substantial increase in PD risk. The key *LRRK2* pathogenic variants include p.R1441C (rs33939927), p.R1441G (rs33939927), p.R1441H (rs33939927), p.G2019S (rs34637584), and p.I2020T (rs35870237). A complete list of the pathogenic or likely pathogenic variants, including reported odds ratios of PD risk, is provided at https://www.ppmi-info.org.

*APOE* genotyping was conducted using baseline DNA samples via standard genotyping protocols. For the PPMI cohort, *APOE* genotyping was based on SNPs rs429358 and rs7412, derived from whole-genome sequencing (WGS) and genome-wide association study (GWAS) arrays. These were supplemented by in-house genotyping at the Indiana University PPMI/Bionet Biorepository using a custom 96-SNP Fluidigm microarray. For the CPP cohort, APOE genotyping was based on SNPs rs429358 and rs7412, derived from Illumina Human Core Exome array data with custom content.

Genotypes were categorized as: any ε2 but excluding ε2/ε4 (ε2/ε2, ε2/ε3), ε3/ε3, and any ε4 (ε3/ε4, ε2/ε4, ε4/ε4). We performed two distinct analyses with *APOE* ε4. In one analysis, we used the convention in the literature to compare any ε4 (ε2/ε4, ε3/ε4, ε4/ε4) to ε3 (ε3/ε3) to ε2 (ε2/ε2, ε2/ε3). In another analysis, we compared any ε4 to all other cases. CSF biomarker data were not available for the CPP cohort. CSF Aβ42 and pTau levels were measured in the PPMI using enzyme-linked immunosorbent assays. These measurements were obtained at the baseline visit and at subsequent follow-up visits; however, for the present study, we only used baseline CSF Aβ42 and pTau in our analyses. Baseline demographics (age, sex, education, and PD duration) were self-reported and verified during clinical assessments. Quantitative variables such as MoCA scores, CSF biomarkers, and age were analyzed as continuous variables.

### Statistical analysis

Multivariate linear mixed-effects models were employed to test two key models, an *APOE* model, and a CSF AD biomarker model, impacting cognitive trajectories over time across diagnostic groups. Time (years from baseline) was treated as a continuous variable, with regression coefficients reflecting annual changes in cognitive function. We analyzed the pTau and Aβ42 groups as continuous data to maximize statistical power. For visual display purposes in Figs. 3 and 4, we divided the groups into three, based on the mean and one SD above and below the mean value for Aβ42 and pTau.

In the *APOE* model, both linear and quadratic time terms were initially included, but for parsimony and robustness, we tested interactions only with the linear time term. This model evaluated the association between *APOE* genotype (ε2, ε3, ε4) and cognitive decline, incorporating interactions between *APOE* genotype, diagnosis, and time. The healthy control (HC) group served as the reference for diagnosis, and APOE ε3 served as the reference for genotype comparisons with ε4 carriers. Fixed effects included diagnosis, APOE genotype, time, and their interactions, along with baseline age, sex, years of education (categorized as ≤12 vs. >12 years to harmonize with CPP data), and PD duration (set to 0 for HC).

The CSF AD biomarker model examined the impact of baseline Aβ42 and pTau levels on cognitive trajectories. Like the *APOE* model, it included interactions between biomarkers, diagnosis, and time, but due to model complexity, only linear time was studied. Additional between-subject effects included baseline age, sex, education as a continuous variable, PD duration, and levodopa equivalent daily dose (LEDD). LEDD was not significant in initial tests and was removed.

Both models utilized a fully crossed mixed design, integrating between-subject factors including diagnosis and either *APOE* genotypes (*APOE* model) or baseline CSF Aβ42 and pTau levels (CSF AD biomarker model). As CSF Aβ42 and pTau biomarkers are continuous measures of AD-related pathological processes that are not exhaustively understood in the context of PD, and as AD-copathology when present in PD is often milder than in patients with isolated AD, we prioritized continuous analyses of these data. Recent analyses from PPMI^46^ based on PPMI-derived Youden-optimized values validated against amyloid PET in PD demonstrated that AD-based cut-offs do not translate well to PD, and identified PD-appropriate thresholds that better correspond to amyloid PET and cognitive decline: PD-specific Aβ42 cut-off: < 945 pg/mL (vs. AD cut-off 683 pg/mL), PD-specific pTau cut-off: > 17 pg/mL (vs. AD cut-off 24 pg/mL). We also explored the effect of PD-thresholded data. Mixed-effects longitudinal analyses were primarily employed, incorporating all relevant fixed effects including two-way and three-way interactions. The primary dependent variable was MoCA. Higher-order interaction terms and covariates were pretested and if nonsignificant, sequentially removed from the models to enhance statistical power for detecting other effects of interest. Random effects included participants and their interaction with time, accounting for individual variability in their intercepts and cognitive trajectories. Significant omnibus tests were followed up with appropriate post hoc tests and contrasts to further investigate the interactions. Effect graphs of model-predicted values of the dependent variable were generated to visually illustrate key findings. Model residuals were examined for conformance to model assumptions. “Type III” F tests are tests of partial effects of a given predictor term (including terms with multiple degrees of freedom) adjusting for all other terms in the model. A sensitivity analysis was also conducted in which PD duration was excluded from the model.

To test whether baseline CSF Aβ42 mediates the association between *APOE ε4* carrier status and longitudinal cognitive decline in sporadic PD, we performed mediation analysis with the mediate () function in the R package ‘mediation’. We applied 5000 bootstrap simulations to estimate the Average Causal Mediation Effect (ACME; indirect effect), Average Direct Effect (ADE), Total Effect (TE), and proportion mediated (PM). The mediator model (Aβ42 ~ *APOE ε4*) was fit with linear regression, and the outcome model (MoCA slope ~ *APOE ε4* + Aβ42) was fit using linear regression after extracting the MoCA slope from a linear mixed-effects model. The outcome model was adjusted for baseline age, sex, years of education, and baseline disease duration, while the mediator model was adjusted for baseline age, sex, and baseline disease duration.

Statistical analyses were performed using R software (Version 2023.12.1, Copyright (C) 2022 by Posit Software, PBC).

### Role of the funding source

The funding source was not involved in the study design, data collection, analysis, interpretation, manuscript preparation, or the decision to submit the paper for publication.

## Supplementary information

Supplementary Information

## Data Availability

The PPMI database is accessible at [https://www.ppmi-info.org/data], and the CPP data can be found at [https://c-path.org/tools-platforms/integrated-parkisons-database/].
