# Supplementary material for: APOE, Aβ42, and tau differentially impact cognitive decline in Sporadic, GBA1 and LRRK2 Parkinson’s disease
Source: NPJ Parkinsons Dis. 2026 Feb 23;12:79. doi: 10.1038/s41531-026-01290-2 (PMC13039544; doi:10.1038/s41531-026-01290-2)
Supplement: Supplementary file 1 — Supplementary Information [file 41531_2026_1290_MOESM1_ESM.docx]

## Supplementary Tables

### Table S1: Demographic and Clinical Baseline Characteristics of Individuals with Parkinson’s Disease: CPP & PPMI Cohorts

| Baseline Characteristics of patients with PD - CPP Dataset | | | | | |
| --- | --- | --- | --- | --- | --- |
|  | **Total (N=1379)** | ***GBA1* (N=29)** | ***LRRK2* (N=3)** | **Sporadic PD (N=1347)** | **P-value** |
| Sex, n (%) |  |  |  |  |  |
| Male | 492 (35.7%) | 11 (37.9%) | 0 (0%) | 481 (35.7%) | 0.63 |
| Female | 887 (64.3%) | 18 (62.1%) | 3 (100%) | 866 (64.3%) |  |
| Age, mean (SD) (Years) | 67.5 (8.7) | 62.6 (10.0) | 58.0 (11.4) | 67.6 (8.6) | 0.02 |
| Education, n (%) |  |  |  |  |  |
| 12 years or less | 432 (31.3%) | 8 (27.6%) | 2 (66.7%) | 422 (31.3%) | 0.59 |
| More than 12 years of higher education | 947 (68.7%) | 21 (72.4%) | 1 (33.3%) | 925 (68.7%) |  |
| Age at Onset of PD mean (SD) (years) | 64.5 (8.9) | 59.5 (10.1) | 56.6 (11.2) | 64.6 (8.8) | 0.02 |
| Data missing | 40 (2.9%) | 0 (0%) | 0 (0%) | 40 (3.0%) |  |
| Duration of PD, mean (SD) (Years) | 3.0 (2.3) | 3.1 (2.0) | 1.39 (0.22) | 3.0 (2.3) | 0.29 |
| Data missing | 40 (2.9%) | 0 (0%) | 0 (0%) | 40 (3.0%) |  |
| *APOE*4 carrier status, n (%) |  |  |  |  |  |
| ε4 | 326 (23.6%) | 7 (24.1%) | 0 (0%) | 319 (23.7%) | 0.82 |
| non ε4 | 1053 (76.4%) | 22 (75.9%) | 3 (100%) | 1028 (76.3%) |  |
| *APOE* Genotype, n (%) |  |  |  |  |  |
| *APOE* ε2 | 192 (13.9%) | 3 (10.3%) | 2 (66.7%) | 187 (13.9%) | 0.28 |
| *APOE* ε3 | 861 (62.4%) | 19 (65.5%) | 1 (33.3%) | 841 (62.4%) |  |
| *APOE* ε4 | 326 (23.6%) | 7 (24.1%) | 0 (0%) | 319 (23.7%) |  |
| MDS-UPDRS Part III Score, mean (SD) | 22.0 (12.1) | 22.0 (12.8) | 32.0 (NA) | 21.9 (12.2) | 0.69 |
| Missing | 1294 (93.8%) | 25 (86.2%) | 2 (66.7%) | 1267 (94.1%) |  |
| Montreal Cognitive Assessment Score, mean (SD) | 25.2 (3.3) | 25.1 (2.7) | 26.7 (2.9) | 25.2 (3.3) | 0.88 |
| Baseline Characteristics of patients with PD - PPMI Dataset | | | | | |
|  | **Total (N=744)** | ***GBA1* (N=95)** | ***LRRK2* (N=163)** | **Sporadic PD (N=486)** | **P-value** |
| Sex, n (%) |  |  |  |  |  |
| Male | 294 (39.5%) | 39 (41.1%) | 85 (52.1%) | 170 (35.0%) | **<0.01** |
| Female | 450 (60.5%) | 56 (58.9%) | 78 (47.9%) | 316 (65.0%) |  |
| Age, mean (SD) (Years) | 63.5 (8.6) | 63.6 (8.8) | 64.6 (8.1) | 63.2 (8.6) | 0.40 |
| Education, n (%) |  |  |  |  |  |
| 12 years or less | 131 (17.6%) | 13 (13.7%) | 39 (23.9%) | 79 (16.3%) | 0.11 |
| More than 12 years of higher education | 613 (82.4%) | 82 (86.3%) | 124 (76.1%) | 407 (83.7%) |  |
| Age at Onset of PD mean (SD) (years) | 62.2 (8.6) | 60.7 (9.2) | 61.8 (8.2) | 62.6 (8.6) | 0.25 |
| Duration of PD, mean (SD) (Years) | 1.3 (1.8) | 2.9 (2.4) | 2.80 (2.1) | 0.6 (0.6) | <0.001 |
| *APOE*4 carrier status, n (%) |  |  |  |  |  |
| ε4 | 186 (25.0%) | 20 (21.1%) | 39 (23.9%) | 127 (26.1%) | 0.75 |
| non ε4 | 558 (75.0%) | 75 (78.9%) | 124 (76.1%) | 359 (73.9%) |  |
| *APOE* Genotype, n (%) |  |  |  |  |  |
| *APOE* ε2 | 101 (13.6%) | 14 (14.7%) | 21 (12.9%) | 66 (13.6%) | 0.97 |
| *APOE* ε3 | 457 (61.4%) | 61 (64.2%) | 103 (63.2%) | 293 (60.3%) |  |
| *APOE* ε4 | 186 (25.0%) | 20 (21.1%) | 39 (23.9%) | 127 (26.1%) |  |
| MDS-UPDRS Part III Score, mean (SD) | 21.8 (9.42) | 25.1 (11.1) | 21.0 (10.2) | 21.5 (8.9) | 0.05 |
| Missing | 58 (7.8%) | 19 (20.0%) | 38 (23.3%) | 1 (0.2%) |  |
| Montreal Cognitive Assessment Score, mean (SD) | 26.7 (2.8) | 26.1 (3.0) | 25.9 (3.3) | 27.1 (2.4) | <0.001 |

Abbreviations: *APOE*, Apolipoprotein E; *GBA1*, Glucocerebrosidase mutation; *LRRK2*, Leucine-rich repeat kinase 2

### Table S2 : Baseline and clinical characteristics of patients with PD included in the *APOE* model stratified by *APOE* genotype

|  | Total (N=2123) | *APOE* ε2 (N=293) | *APOE* ε3 (N=1318) | *APOE* ε4 (N=512) | P-value |
| --- | --- | --- | --- | --- | --- |
| Age at Baseline (years) (mean (SD)) | 66.1 (8.8) | 66.3 (9.0) | 66.3 (8.8) | 65.7 (8.8) | 0.59 |
| Sex = Male (%) | 1337 (63.0%) | 175 (59.7%) | 839 (63.7%) | 323 (63.1%) | 0.66 |
| Education |  |  |  |  |  |
| 12 years or less | 563 (26.5%) | 74 (25.3%) | 349 (26.5%) | 140 (27.3%) | 0.94 |
| more than 12 years or higher education | 1560 (73.5%) | 219 (74.7%) | 969 (73.5%) | 372 (72.7%) |  |
| Age at Onset of PD (years) (mean (SD)) | 63.7 (8.8) | 63.9 (9.1) | 63.8 (8.9) | 63.2 (8.6) | 0.43 |
| Data Missing | 40 (1.9%) | 5 (1.7%) | 27 (2.0%) | 8 (1.6%) |  |
| Duration of PD (years) (mean (SD)) | 2.43 (2.3) | 2.41 (2.2) | 2.43 (2.3) | 2.46 (2.3) | 0.10 |
| Data Missing | 40 (1.9%) | 5 (1.7%) | 27 (2.0%) | 8 (1.6%) |  |
|  |  |  |  |  |  |
| MDS-UPDRS Part III Score (mean (SD)) | 21.9 (9.7) | 21.9 (10.5) | 22.3 (9.9) | 20.8 (8.9) | 0.54 |
| Data Missing | 1352 (63.7%) | 185 (63.1%) | 845 (64.1%) | 322 (62.9%) |  |
| Montreal Cognitive Assessment Score (mean (SD)) | 25.7 (3.2) | 25.7 (3.4) | 25.7 (3.1) | 25.7 (3.4) | 0.96 |

Abbreviations: PD, Parkinson’s Disease; *APOE*, Apolipoprotein E; MDS-UPDRS, MDS-Unified Parkinson's Disease Rating Scale; SD, standard deviation

Results are presented as number (%) or mean (SD). Percentages may not sum to 100 due to rounding. *APOE* genotypes are categorized as follows: *APOE* ε3 (two copies of the ε3 allele), *APOE* ε2 (ε2/ε2 or ε2/ε3), and *APOE* ε4 (ε3/ε4, ε2/ε4, or ε4/ε4)

###

### Table S3: Baseline and clinical characteristics of patients and healthy controls included in the CSF AD biomarker model stratified by diagnosis

|  | Overall | Healthy Control | Sporadic | *GBA1* | *LRRK2* | p value |
| --- | --- | --- | --- | --- | --- | --- |
| N | **606** | **131** | **304** | **54** | **117** |  |
| **Age at Baseline (mean (SD))** | 62.9 (8.7) | 62.5 (9.5) | 62.8 (8.3) | 62.7 (8.7) | 63.8 (8.7) | 0.53 |
| **Sex= Male (%)** | 376 (62.0%) | 84 (64.1%) | 200 (65.8%) | 33 (61.1%) | 59 (50.4%) | **<0.01** |
| **Years of Education (mean (SD))** | 15.7 (3.4) | 16.1 (2.9) | 15.4 (3.0) | 16.3 (3.9) | 15.4 (4.4) | 0.067 |
| **Duration of PD (years) (mean (SD))** | 1.4 (2.4) | - | 1.3 (2.6) | 2.7 (2.2) | 2.7 (2.1) | **<0.001** |
| **Data Missing** | 3 (0.5%) | - | 0 (0%) | 0 (0%) | 3 (2.6%) |  |
| ***APOE*_class1 (%)** |  |  |  |  |  | 0.70 |
| ***APOE*3** | 379 (62.5%) | 82 (62.6%) | 184 (60.5%) | 35 (64.8%) | 78 (66.7%) |  |
| ***APOE*2** | 80 (13.2%) | 12 (9.2%) | 43 (14.1%) | 8 (14.8%) | 17 (14.5%) |  |
| ***APOE*4** | 147 (24.3%) | 37 (28.2%) | 77 (25.3%) | 11 (20.4%) | 22 (18.8%) |  |
| **MDS-UPDRS Part III Score (mean (SD))** | 16.5 (12.0) | 1.4 (2.4) | 20.5 (9.4) | 24.9 (10.6) | 20.8 (10.8) | **<0.001** |
| **Data Missing** | 36 (5.9%) | 0 (0%) | 0 (0%) | 8 (14.8%) | 28 (23.9%) |  |
| **Montreal Cognitive Assessment Score (mean (SD))** | 27.1 (2.4) | 28.2 (1.1) | 27.1 (2.3) | 26.4 (2.5) | 26.1 (2.9) | **<0.001** |
| **pTau at Baseline (mean (SD))** | 14.5 (5.8) | 16.2 (8.3) | 14.0 (4.6) | 13.6 (5.9) | 14.1 (4.7) | **<0.001** |
| **Tau at Baseline (mean (SD))** | 169 (55.8) | 183 (71.6) | 165 (47.7) | 159 (62.2) | 165 (49.4) | **<0.001** |
| Aβ42 at Baseline (mean (SD)) | 857 (274) | 887 (264) | 865 (277) | 739 (270) | 858 (267) | <0.01 |

Abbreviations: PD, Parkinson’s Disease; *APOE*, Apolipoprotein E; CSF, Cerebrospinal fluid; MDS-UPDRS, MDS-Unified Parkinson's Disease Rating Scale; *GBA1*, Glucocerebrosidase mutation; *LRRK2*, Leucine-rich repeat kinase 2; SD, standard deviation

Results are presented as number (%) or mean (SD). Percentages may not sum to 100 due to rounding. *APOE* genotypes are categorized as follows: *APOE* ε3 (two copies of the ε3 allele), *APOE* ε2 (ε2/ε2 or ε2/ε3), and *APOE* ε4 (ε3/ε4, ε2/ε4, or ε4/ε4)

### Table S4: Baseline Characteristics of subjects with PD included in the CSF AD biomarker model stratified by *APOE* genotype

|  | Overall | *APOE* ε3 | *APOE* ε2 | *APOE* ε4 | p |
| --- | --- | --- | --- | --- | --- |
| n | **475** | **297** | **68** | **110** |  |
| Age at Baseline (mean (SD)) | 63.0 (8.4) | 63.3 (8.3) | 63.0 (8.3) | 62.4 (9.0) | 0.68 |
| Sex = Male (%) | 292 (61.5) | 180 (60.6) | 38 (55.9) | 74 (67.3) | 0.28 |
| Years of Education (mean (SD)) | 15.5 (3.5) | 15.3 (3.6) | 16.2 (3.3) | 15.8 (3.4) | 0.12 |
| Duration of PD (mean (SD)) | 1.8 (2.5) | 1.8 (2.4) | 1.9 (2.2) | 1.9 (3.0) | 0.83 |
| Data Missing | 3 (0.6%) | 2 (0.7%) | 1 (1.5%) | 0 (0%) |  |
| MDS-UPDRS Part III Score (mean (SD)) | 21.0 (9.9) | 21.4 (10.0) | 20.4 (10.7) | 20.2 (9.0) | 0.35 |
| Data Missing | 36 (7.6%) | 24 (8.1%) | 5 (7.4%) | 7 (6.4%) |  |
| Montreal Cognitive Assessment Score (mean (SD)) | 26.8(2.5) | 26.8 (2.3) | 26.7 (3.1) | 26.8 (2.6) | 0.97 |
| Baseline pTau (mean (SD)) | 14.0 (4.8) | 13.9 (4.7) | 13.8 (4.7) | 14.4 (5.1) | 0.55 |
| Baseline Aβ42 (mean (SD)) | 849.0 (276.0) | 897.2 (271.2) | 834.8 (283.8) | 727.5 (246.1) | **<0.001** |

Abbreviations: PD, Parkinson’s Disease; *APOE*, Apolipoprotein E; MDS_UPDRS, MDS-Unified Parkinson's Disease Rating Scale; \; SD, standard deviation. Results are presented as number (%) or mean (SD). Percentages may not sum to 100 due to rounding. *APOE* genotypes are categorized as follows: *APOE* ε3 (two copies of the ε3 allele), *APOE* ε2 (ε2/ε2 or ε2/ε3), and *APOE* ε4 (ε3/ε4, ε2/ε4, or ε4/ε4)

###

### Table S5: Type III ANOVA Results for Mixed Effects Model – *APOE* Model (*APOE* ε4 vs *APOE* ε3, *APOE* ε2 vs *APOE* ε3)

|  | Sum Sq | Mean Sq | NumDF | DenDF | F value | Pr(>F) |
| --- | --- | --- | --- | --- | --- | --- |
| Diagnosis | 62.496 | 20.832 | 3 | 2018.877 | 6.545 | **<0.0001** |
| Time | 360.867 | 360.867 | 1 | 1223.499 | 113.381 | **<0.0001** |
| *APOE* | 6.440 | 3.220 | 2 | 2021.718 | 1.012 | 0.36 |
| Age at Baseline | 656.603 | 656.603 | 1 | 2158.970 | 206.299 | **<0.0001** |
| I (Time ^2) | 77.337 | 77.337 | 1 | 8398.588 | 24.299 | **<0.0001** |
| Education | 417.662 | 417.662 | 1 | 2205.802 | 131.226 | **<0.0001** |
| Sex | 127.879 | 127.879 | 1 | 2211.477 | 40.178 | **<0.0001** |
| Duration of PD at Baseline | 44.398 | 44.398 | 1 | 2296.436 | 13.950 | **<0.0001** |
| Diagnosis x Time | 89.911 | 29.970 | 3 | 865.610 | 9.416 | **<0.0001** |
| Diagnosis x *APOE* | 7.602 | 1.267 | 6 | 2014.721 | 0.398 | 0.88 |
| *APOE* x Time | 14.604 | 7.302 | 2 | 886.562 | 2.294 | 0.10 |
| Age at Baseline x Time | 533.651 | 533.651 | 1 | 1114.675 | 167.668 | **<0.0001** |
| Education x Time | 27.558 | 27.558 | 1 | 1176.599 | 8.659 | **<0.01** |
| Diagnosis x *APOE* x Time | 66.582 | 11.097 | 6 | 867.116 | 3.487 | **<0.01** |

Abbreviations: *APOE*, Apolipoprotein E; ANOVA, Analysis of Variance; NumDF, Degrees of freedom of numerator; DenDF, Degrees of freedom of denominator; Time^2 is the quadratic time

### Table S6: Pairwise Contrasts of Estimated Marginal Means by Diagnosis and *APOE* subtype at different time points

| Contrast | Years from Baseline | Diagnosis | Estimate | SE | df | z.ratio | p.value |
| --- | --- | --- | --- | --- | --- | --- | --- |
| *APOE*3 - *APOE*2 | 1.49 | Healthy Control | -0.216 | 0.583 | Inf | -0.370 | 0.93 |
| *APOE*3 - *APOE*4 | 1.49 | Healthy Control | 0.424 | 0.443 | Inf | 0.958 | 0.60 |
| *APOE*2 - *APOE*4 | 1.49 | Healthy Control | 0.640 | 0.655 | Inf | 0.977 | 0.59 |
| *APOE*3 - *APOE*2 | 1.49 | *GBA1* | -0.026 | 0.722 | Inf | -0.036 | 0.10 |
| *APOE*3 - *APOE*4 | 1.49 | *GBA1* | -0.141 | 0.605 | Inf | -0.234 | 0.97 |
| *APOE*2 - *APOE*4 | 1.49 | *GBA1* | -0.116 | 0.839 | Inf | -0.138 | 0.99 |
| *APOE*3 - *APOE*2 | 1.49 | *LRRK2* | 0.181 | 0.617 | Inf | 0.293 | 0.95 |
| *APOE*3 - *APOE*4 | 1.49 | *LRRK2* | 0.798 | 0.511 | Inf | 1.561 | 0.26 |
| *APOE*2 - *APOE*4 | 1.49 | *LRRK2* | 0.617 | 0.709 | Inf | 0.870 | 0.65 |
| *APOE*3 - *APOE*2 | 1.49 | Sporadic PD | -0.118 | 0.191 | Inf | -0.618 | 0.81 |
| *APOE*3 - *APOE*4 | 1.49 | Sporadic PD | 0.433 | 0.154 | Inf | 2.817 | **0.01** |
| *APOE*2 - *APOE*4 | 1.49 | Sporadic PD | 0.551 | 0.216 | Inf | 2.545 | **0.03** |
| *APOE*3 - *APOE*2 | 2.99 | Healthy Control | -0.055 | 0.667 | Inf | -0.083 | 0.10 |
| *APOE*3 - *APOE*4 | 2.99 | Healthy Control | 0.590 | 0.504 | Inf | 1.169 | 0.47 |
| *APOE*2 - *APOE*4 | 2.99 | Healthy Control | 0.645 | 0.749 | Inf | 0.861 | 0.67 |
| *APOE*3 - *APOE*2 | 2.99 | *GBA1* | 0.208 | 0.832 | Inf | 0.250 | 0.97 |
| *APOE*3 - *APOE*4 | 2.99 | *GBA1* | -0.318 | 0.705 | Inf | -0.451 | 0.89 |
| *APOE*2 - *APOE*4 | 2.99 | *GBA1* | -0.527 | 0.971 | Inf | -0.542 | 0.85 |
| *APOE*3 - *APOE*2 | 2.99 | *LRRK2* | 0.605 | 0.699 | Inf | 0.866 | 0.66 |
| *APOE*3– *APOE*4 | 2.99 | *LRRK2* | 1.031 | 0.591 | Inf | 1.744 | 0.19 |
| *APOE*2 - *APOE*4 | 2.99 | *LRRK2* | 0.426 | 0.810 | Inf | 0.526 | 0.86 |
| *APOE*3 - *APOE*2 | 2.99 | Sporadic PD | -0.307 | 0.222 | Inf | -1.380 | 0.35 |
| *APOE*3 - *APOE*4 | 2.99 | Sporadic PD | 0.810 | 0.179 | Inf | 4.531 | **<0.0001** |
| *APOE*2 - *APOE*4 | 2.99 | Sporadic PD | 1.117 | 0.252 | Inf | 4.432 | **<0.0001** |
| *APOE*3 - *APOE*2 | 10 | Healthy Control | 0.692 | 1.415 | Inf | 0.489 | 0.88 |
| *APOE*3 - *APOE*4 | 10 | Healthy Control | 1.360 | 1.045 | Inf | 1.301 | 0.39 |
| *APOE*2 - *APOE*4 | 10 | Healthy Control | 0.669 | 1.576 | Inf | 0.425 | 0.91 |
| *APOE*3 - *APOE*2 | 10 | *GBA1* | 1.298 | 1.835 | Inf | 0.707 | 0.76 |
| *APOE*3 - *APOE*4 | 10 | *GBA1* | -1.141 | 1.610 | Inf | -0.709 | 0.76 |
| *APOE*2 - *APOE*4 | 10 | *GBA1* | -2.439 | 2.168 | Inf | -1.125 | 0.50 |
| *APOE*3 - *APOE*2 | 10 | *LRRK2* | 2.581 | 1.451 | Inf | 1.779 | 0.18 |
| *APOE*3 - *APOE*4 | 10 | *LRRK2* | 2.116 | 1.301 | Inf | 1.627 | 0.23 |
| *APOE*2 - *APOE*4 | 10 | *LRRK2* | -0.465 | 1.725 | Inf | -0.269 | 0.96 |
| *APOE*3 - *APOE*2 | 10 | Sporadic PD | -1.183 | 0.511 | Inf | -2.317 | 0.05 |
| *APOE*3 - *APOE*4 | 10 | Sporadic PD | 2.569 | 0.416 | Inf | 6.175 | **<0.0001** |
| *APOE*2 - *APOE*4 | 10 | Sporadic PD | 3.752 | 0.584 | Inf | 6.420 | **<0.0001** |

Pairwise contrasts of estimated marginal means (EMMs) for *APOE* genotypes within each diagnosis across different follow-up times. The time variable (‘Years from Baseline’) represents different follow-up durations: 1.49 years is the earliest time point where a significant difference is observed between *APOE*4 and *APOE*3, 2.99 years represents the model estimated mean follow-up time in the dataset, and 10 years is the maximum recorded follow-up duration. Statistical significance is adjusted using Tukey’s method for multiple comparisons.

### Table S7 : Type III ANOVA Results for Mixed Effects Model – *APOE*4 Model (*APOE* ε4 Carriers vs ε4 non-carriers)

|  | Sum Sq | Mean Sq | NumDF | DenDF | F value | Pr(>F) |
| --- | --- | --- | --- | --- | --- | --- |
| Diagnosis | 56.337 | 18.779 | 3 | 2043.126 | 5.896 | **<0.001** |
| Time | 364.640 | 364.640 | 1 | 1223.600 | 114.492 | **<0.0001** |
| *APOE*4 | 4.7498 | 4.750 | 1 | 2046.425 | 1.491 | 0.22 |
| Age at Baseline | 657.157 | 657.157 | 1 | 2162.794 | 206.338 | **<0.0001** |
| Education | 419.299 | 419.299 | 1 | 2210.072 | 131.654 | **<0.0001** |
| I(Time^2) | 78.488 | 78.488 | 1 | 8401.728 | 24.644 | **<0.0001** |
| Sex | 127.672 | 127.672 | 1 | 2215.440 | 40.087 | **<0.0001** |
| Duration of PD at Baseline | 44.516 | 44.516 | 1 | 2300.414 | 13.977 | **<0.0001** |
| Diagnosis x Time | 92.537 | 30.846 | 3 | 890.202 | 9.685 | **<0.0001** |
| Diagnosis x *APOE*4 | 4.862 | 1.620 | 3 | 2033.824 | 0.509 | 0.68 |
| Time x *APOE*4 | 6.546 | 6.546 | 1 | 924.780 | 2.055 | 0.15 |
| Time x Age at Baseline | 531.859 | 531.859 | 1 | 1113.399 | 166.996 | **<0.0001** |
| Time x Education | 26.631 | 26.631 | 1 | 1176.716 | 8.362 | **<0.01** |
| Diagnosis x Time x *APOE*4 | 32.167 | 10.722 | 3 | 887.124 | 3.367 | **0.02** |

Abbreviations: *APOE*, Apolipoprotein E; ANOVA, Analysis of Variance; NumDF, Degrees of freedom of numerator; DenDF, Degrees of freedom of denominator; Time^2 is the quadratic time

### Table S8: Pairwise Contrasts of Estimated Marginal Means in PD subgroups and Healthy controls in *APOE*4 carriers vs non-carriers at different time points

| Contrast | Diagnosis | Years from Baseline | Estimate | SE | df | z.ratio | p.value |
| --- | --- | --- | --- | --- | --- | --- | --- |
| non e4 - e4 | Healthy Control | 0.97 | 0.410 | 0.421 | Inf | 0.974 | 0.33 |
| non e4 - e4 | *GBA1* | 0.97 | -0.059 | 0.575 | Inf | -0.103 | 0.92 |
| non e4 - e4 | *LRRK2* | 0.97 | 0.708 | 0.484 | Inf | 1.462 | 0.14 |
| non e4 - e4 | Sporadic PD | 0.97 | 0.312 | 0.146 | Inf | 2.136 | **0.03** |
| non e4 - e4 | Healthy Control | 2.99 | 0.600 | 0.493 | Inf | 1.217 | 0.22 |
| non e4 - e4 | *GBA1* | 2.99 | -0.358 | 0.689 | Inf | -0.520 | 0.60 |
| non e4 - e4 | *LRRK2* | 2.99 | 0.910 | 0.576 | Inf | 1.579 | 0.11 |
| non e4 - e4 | Sporadic PD | 2.99 | 0.865 | 0.174 | Inf | 4.965 | **<0.0001** |
| non e4 - e4 | Healthy Control | 10 | 1.257 | 1.023 | Inf | 1.229 | 0.22 |
| non e4 - e4 | *GBA1* | 10 | -1.392 | 1.573 | Inf | -0.885 | 0.38 |
| non e4 - e4 | *LRRK2* | 10 | 1.607 | 1.270 | Inf | 1.265 | 0.21 |
| non e4 - e4 | Sporadic PD | 10 | 2.778 | 0.406 | Inf | 6.839 | **<0.0001** |

Pairwise contrasts of estimated marginal means (EMMs) for *APOE*4 carriers and non-carriers within each diagnosis across different follow-up times are shown. The time variable (‘Years from Baseline’) represents different follow-up durations: 0.97 years is the earliest time point where a significant difference is observed between *APOE*4 and *APOE*3, 2.99 years represents the model estimated mean follow-up time in the dataset, and 10 years is the maximum recorded follow-up duration. Statistical significance is adjusted using Tukey’s method for multiple comparisons.

### Table S9: Unstandardized & Standardized regression coefficients (β) for the fixed effects on cognitive decline (MoCA) over time in the *APOE*4 model

|  | Unstandardized partial regression coefficients | | | Standardized partial regression coefficients | | | |
| --- | --- | --- | --- | --- | --- | --- | --- |
| Factors | **Estimates** | **95% CI** | **p** | | **Estimates** | **95% CI** | **p** |
| (Intercept) | 32.45 | 31.51 – 33.39 | **<0.001** | | 26.24 | 25.68 – 26.80 | **<0.001** |
| Reference: Diagnosis [Healthy Control] | | | | | | | |
| Diagnosis [*GBA1*] | -1.28 | -1.96 – -0.60 | **<0.001** | | -2.88 | -3.68 – -2.09 | **<0.001** |
| Diagnosis [*LRRK2*] | -0.90 | -1.52 – -0.28 | **0.004** | | -0.92 | -1.64 – -0.19 | **0.013** |
| Diagnosis [Sporadic PD] | -1.04 | -1.48 – -0.60 | **<0.001** | | -1.31 | -1.83 – -0.79 | **<0.001** |
| Time | 1.38 | 1.13 – 1.63 | **<0.001** | | -0.26 | -0.55 – 0.02 | 0.07 |
| *APOE*4 [e4] | -0.32 | -1.13 – 0.49 | 0.44 | | -0.60 | -1.56 – 0.37 | 0.22 |
| Age at Baseline | -0.09 | -0.10 – -0.08 | **<0.001** | | -1.38 | -1.51 – -1.25 | **<0.001** |
| Education [more than 12years or higher education] | 1.50 | 1.25 – 1.76 | **<0.001** | | 1.81 | 1.50 – 2.11 | **<0.001** |
| Time^2 | -0.01 | -0.02 – -0.01 | **<0.001** | | -0.10 | -0.14 – -0.06 | **<0.001** |
| Sex [Male] | -0.73 | -0.95 – -0.50 | **<0.001** | | -0.73 | -0.95 – -0.50 | **<0.001** |
| Duration of PD at Baseline | -0.10 | -0.15 – -0.05 | **<0.001** | | -0.20 | -0.30 – -0.09 | **<0.001** |
| Diagnosis [*GBA1*] × Time | -0.54 | -0.70 – -0.37 | **<0.001** | | -1.39 | -1.82 – -0.96 | **<0.001** |
| Diagnosis [*LRRK2*] × Time | -0.01 | -0.15 – 0.14 | 0.94 | | -0.01 | -0.38 – 0.35 | 0.94 |
| Diagnosis [Sporadic PD] × Time | -0.09 | -0.19 – 0.01 | 0.08 | | -0.23 | -0.50 – 0.03 | 0.08 |
| Diagnosis [*GBA1*] × *APOE*4 [e4] | 0.23 | -1.16 – 1.63 | 0.74 | | 0.95 | -0.70 – 2.61 | 0.26 |
| Diagnosis [*LRRK2*] × *APOE*4 [e4] | -0.29 | -1.54 – 0.95 | 0.65 | | -0.31 | -1.79 – 1.17 | 0.68 |
| Diagnosis [Sporadic PD] ×*APOE*4 [e4] | 0.27 | -0.59 – 1.14 | 0.54 | | -0.26 | -1.28 – 0.76 | 0.62 |
| Time × *APOE*4 [e4] | -0.09 | -0.28 – 0.09 | 0.33 | | -0.24 | -0.73 – 0.24 | 0.33 |
| Time × Age at Baseline | -0.02 | -0.02 – -0.02 | **<0.001** | | -0.49 | -0.57 – -0.42 | **<0.001** |
| Time × Education [more than 12 years or higher education] | 0.10 | 0.03 – 0.17 | **<0.01** | | 0.26 | 0.09 – 0.44 | **<0.01** |
| (Diagnosis [*GBA1*] × Time) ×*APOE*4 [e4] | 0.24 | -0.11 – 0.60 | 0.18 | | 0.62 | -0.29 – 1.54 | 0.18 |
| (Diagnosis [*LRRK2*] × Time) × *APOE*4 [e4] | -0.01 | -0.31 – 0.30 | 0.97 | | -0.01 | -0.80 – 0.77 | 0.97 |
| (Diagnosis [Sporadic PD] ×Time) × *APOE*4 [e4] | -0.18 | -0.38 – 0.02 | 0.08 | | -0.46 | -0.99 – 0.06 | 0.08 |

Abbreviations: *APOE*, Apolipoprotein E; *GBA1*, Glucocerebrosidase mutation; *LRRK2*, Leucine-rich repeat kinase 2; Time^2 is the quadratic time Education [1] = Education [more than 12 years or higher education

### Table S10: Type III ANOVA Results for Mixed Effects *APOE* Model with Cohort level inclusion in the model

|  | **Sum Sq** | **Mean Sq** | **NumDF** | **DenDF** | **F value** | **Pr(>F)** |
| --- | --- | --- | --- | --- | --- | --- |
| **Diagnosis** | 73.189 | 24.396 | 3 | 2066.995 | 7.654 | **<0.001** |
| **Time** | 362.413 | 362.413 | 1 | 1254.103 | 113.701 | **<0.001** |
| ***APOE*4** | 5.012 | 5.012 | 1 | 2082.300 | 1.572 | 0.21 |
| **Age at Baseline** | 620.703 | 620.703 | 1 | 2209.996 | 194.735 | **<0.001** |
| **Education** | 396.297 | 396.297 | 1 | 2254.887 | 124.331 | **<0.001** |
| **I (Time ^2)** | 68.047 | 68.047 | 1 | 8564.804 | 21.349 | **<0.001** |
| **Sex** | 135.419 | 135.419 | 1 | 2254.550 | 42.485 | **<0.001** |
| **Cohort** | 137.842 | 137.842 | 1 | 2217.007 | 43.246 | **<0.001** |
| **Diagnosis x Time** | 89.097 | 29.699 | 3 | 904.429 | 9.318 | **<0.001** |
| **Diagnosis x *APOE*4** | 5.638 | 1.879 | 3 | 2069.165 | 0.590 | 0.62 |
| **Time x *APOE*4** | 6.497 | 6.497 | 1 | 939.704 | 2.038 | 0.15 |
| **Time x Age at Baseline** | 534.870 | 534.870 | 1 | 1141.150 | 167.806 | **<0.001** |
| **Time x Education** | 27.504 | 27.504 | 1 | 1203.354 | 8.629 | **<0.001** |
| **Diagnosis x Time x *APOE*4** | 31.426 | 10.475 | 3 | 900.903 | 3.286 | **0.02** |

###

Table S11: Difference in annual rate of MoCA decline associated with APOE ε4 carrier status across PD subgroups

| **PD subgroup** | **Contrast** | **Difference in annual**  **MoCA decline (points/year)** | **95% CI** | **p value** |
| --- | --- | --- | --- | --- |
| ***GBA1*** PD | ***APOE ε4*** − non-carrier | -0.15 | -0.47 to 0.18 | 0.38 |
| ***LRRK2*** PD | ***APOE ε4*** − non-carrier | 0.11 | -0.15 to 0.36 | 0.42 |
| Sporadic PD | ***APOE ε4*** − non-carrier | 0.28 | 0.2 to 0.37 | <**0.001** |

###

### Table S12: Type III ANOVA Results for Mixed Effects CSF AD Biomarker Model

|  | Sum Sq | Mean Sq | NumDF | DenDF | F value | Pr(>F) |
| --- | --- | --- | --- | --- | --- | --- |
| Diagnosis | 5.623 | 1.874 | 3 | 619.033 | 0.670 | 0.57 |
| Time | 12.334 | 12.334 | 1 | 347.208 | 4.412 | **0.04** |
| Baseline pTau | 4.825 | 4.825 | 1 | 655.575 | 1.726 | 0.19 |
| Aβ42 at Baseline | 12.973 | 12.973 | 1 | 570.737 | 4.640 | **0.03** |
| Age at Baseline | 124.427 | 124.427 | 1 | 567.533 | 44.503 | **<0.0001** |
| Education | 48.893 | 48.893 | 1 | 578.100 | 17.487 | **<0.0001** |
| Sex | 18.046 | 18.046 | 1 | 567.335 | 6.454 | **0.01** |
| Duration of PD at Baseline | 8.973 | 8.973 | 1 | 602.324 | 3.209 | 0.07 |
| Diagnosis x Time | 44.395 | 14.798 | 3 | 480.919 | 5.293 | **0.001** |
| Diagnosis x pTau at Baseline | 2.709 | 0.903 | 3 | 636.512 | 0.323 | 0.81 |
| Time x pTau at Baseline | 49.580 | 49.580 | 1 | 669.885 | 17.733 | **<0.0001** |
| Time x Aβ42 at Baseline | 85.500 | 85.500 | 1 | 335.249 | 30.580 | **<0.0001** |
| Time x Age at Baseline | 110.281 | 110.281 | 1 | 322.189 | 39.444 | **<0.0001** |
| Time x Years of Education | 18.901 | 18.901 | 1 | 352.443 | 6.760 | **0.01** |
| Time x Duration of PD at Baseline | 23.517 | 23.517 | 1 | 393.155 | 8.411 | **<0.01** |
| Diagnosis x Time x pTau at Baseline | 85.845 | 28.615 | 3 | 537.182 | 10.235 | **<0.0001** |

Abbreviations: *APOE*, Apolipoprotein E;, ANOVA, Analysis of Variance; NumDF, Degrees of freedom of numerator; DenDF, Degrees

of freedom of denominator, Aβ42 – Amyloid beta42, Time^2 is the quadratic time

Table S13: Type III ANOVA Results for Mixed Effects dichotomized CSF Abeta42 Biomarker Model

|  | **Sum Sq** | **Mean Sq** | **NumDF** | **DenDF** | **F value** | **Pr(>F)** |
| --- | --- | --- | --- | --- | --- | --- |
| **Diagnosis** | 46.0745 | 15.3582 | 3 | 645.9945 | 5.5321 | 0.0009 |
| **Time** | 68.0552 | 68.0552 | 1 | 357.2259 | 24.5139 | 0.0000 |
| **Abeta group** | 24.4376 | 24.4376 | 1 | 607.2129 | 8.8026 | 0.0031 |
| **Age at Baseline** | 176.4609 | 176.4609 | 1 | 622.4083 | 63.5622 | 0.0000 |
| **Years of Education** | 46.5586 | 46.5586 | 1 | 627.4850 | 16.7707 | 0.0000 |
| **Sex** | 24.2602 | 24.2602 | 1 | 616.3255 | 8.7387 | 0.0032 |
| **Duration of PD** | 6.9284 | 6.9284 | 1 | 654.2440 | 2.4957 | 0.1146 |
| **Diagnosis x Time** | 86.1378 | 28.7126 | 3 | 418.4028 | 10.3424 | 0.0000 |
| **Time x Abeta group** | 13.2128 | 13.2128 | 1 | 323.3415 | 4.7593 | 0.0299 |
| **Timex Age at Baseline** | 226.6748 | 226.6748 | 1 | 353.6792 | 81.6496 | 0.0000 |
| **Time x Years of Education** | 11.2131 | 11.2131 | 1 | 369.3294 | 4.0390 | 0.0452 |
| **Time x Duration of PD** | 18.6173 | 18.6173 | 1 | 403.3207 | 6.7060 | 0.0100 |

Table S14: Pairwise Contrasts of Estimated Marginal Means in PD subgroups and Healthy controls in Normal vs Low– PD Cut-offs at different time points

| **Contrast** | **Diagnosis** | **Time** | **Estimate** | **SE** | **df** | **z.ratio** | **p.value** |
| --- | --- | --- | --- | --- | --- | --- | --- |
| Normal Abeta - Low Abeta | Healthy Control | 1 | 0.600 | 0.179 | Inf | 3.342 | 0.001 |
| Normal Abeta - Low Abeta | Sporadic PD | 1 | 0.600 | 0.179 | Inf | 3.342 | 0.001 |
| Normal Abeta - Low Abeta | GBA1 PD | 1 | 0.600 | 0.179 | Inf | 3.342 | 0.001 |
| Normal Abeta - Low Abeta | LRRK2 PD | 1 | 0.600 | 0.179 | Inf | 3.342 | 0.001 |
| Normal Abeta - Low Abeta | Healthy Control | 2 | 0.677 | 0.190 | Inf | 3.570 | <0·001 |
| Normal Abeta - Low Abeta | Sporadic PD | 2 | 0.677 | 0.190 | Inf | 3.570 | <0·001 |
| Normal Abeta - Low Abeta | GBA1 PD | 2 | 0.677 | 0.190 | Inf | 3.570 | <0·001 |
| Normal Abeta - Low Abeta | LRRK2 PD | 2 | 0.677 | 0.190 | Inf | 3.570 | <0·001 |
| Normal Abeta - Low Abeta | Healthy Control | 3 | 0.755 | 0.206 | Inf | 3.670 | <0·001 |
| Normal Abeta - Low Abeta | Sporadic PD | 3 | 0.755 | 0.206 | Inf | 3.670 | <0·001 |
| Normal Abeta - Low Abeta | GBA1 PD | 3 | 0.755 | 0.206 | Inf | 3.670 | <0·001 |
| Normal Abeta - Low Abeta | LRRK2 PD | 3 | 0.755 | 0.206 | Inf | 3.670 | <0·001 |
| Normal Abeta - Low Abeta | Healthy Control | 4 | 0.833 | 0.226 | Inf | 3.680 | <0·001 |
| Normal Abeta - Low Abeta | Sporadic PD | 4 | 0.833 | 0.226 | Inf | 3.680 | <0·001 |
| Normal Abeta - Low Abeta | GBA1 PD | 4 | 0.833 | 0.226 | Inf | 3.680 | <0·001 |
| Normal Abeta - Low Abeta | LRRK2 PD | 4 | 0.833 | 0.226 | Inf | 3.680 | <0·001 |
| Normal Abeta - Low Abeta | Healthy Control | 5 | 0.911 | 0.250 | Inf | 3.638 | <0·001 |
| Normal Abeta - Low Abeta | Sporadic PD | 5 | 0.911 | 0.250 | Inf | 3.638 | <0·001 |
| Normal Abeta - Low Abeta | GBA1 PD | 5 | 0.911 | 0.250 | Inf | 3.638 | <0·001 |
| Normal Abeta - Low Abeta | LRRK2 PD | 5 | 0.911 | 0.250 | Inf | 3.638 | <0·001 |
| Normal Abeta - Low Abeta | Healthy Control | 6 | 0.988 | 0.277 | Inf | 3.571 | <0·001 |
| Normal Abeta - Low Abeta | Sporadic PD | 6 | 0.988 | 0.277 | Inf | 3.571 | <0·001 |
| Normal Abeta - Low Abeta | GBA1 PD | 6 | 0.988 | 0.277 | Inf | 3.571 | <0·001 |
| Normal Abeta - Low Abeta | LRRK2 PD | 6 | 0.988 | 0.277 | Inf | 3.571 | <0·001 |
| Normal Abeta - Low Abeta | Healthy Control | 7 | 1.066 | 0.305 | Inf | 3.494 | <0·001 |
| Normal Abeta - Low Abeta | Sporadic PD | 7 | 1.066 | 0.305 | Inf | 3.494 | <0·001 |
| Normal Abeta - Low Abeta | GBA1 PD | 7 | 1.066 | 0.305 | Inf | 3.494 | <0·001 |
| Normal Abeta - Low Abeta | LRRK2 PD | 7 | 1.066 | 0.305 | Inf | 3.494 | <0·001 |
| Normal Abeta - Low Abeta | Healthy Control | 8 | 1.144 | 0.335 | Inf | 3.415 | 0.001 |
| Normal Abeta - Low Abeta | Sporadic PD | 8 | 1.144 | 0.335 | Inf | 3.415 | 0.001 |
| Normal Abeta - Low Abeta | GBA1 PD | 8 | 1.144 | 0.335 | Inf | 3.415 | 0.001 |
| Normal Abeta - Low Abeta | LRRK2 PD | 8 | 1.144 | 0.335 | Inf | 3.415 | 0.001 |
| Normal Abeta - Low Abeta | Healthy Control | 9 | 1.222 | 0.366 | Inf | 3.340 | 0.001 |
| Normal Abeta - Low Abeta | Sporadic PD | 9 | 1.222 | 0.366 | Inf | 3.340 | 0.001 |
| Normal Abeta - Low Abeta | GBA1 PD | 9 | 1.222 | 0.366 | Inf | 3.340 | 0.001 |
| Normal Abeta - Low Abeta | LRRK2 PD | 9 | 1.222 | 0.366 | Inf | 3.340 | 0.001 |
| Normal Abeta - Low Abeta | Healthy Control | 10 | 1.300 | 0.397 | Inf | 3.270 | 0.001 |
| Normal Abeta - Low Abeta | Sporadic PD | 10 | 1.300 | 0.397 | Inf | 3.270 | 0.001 |
| Normal Abeta - Low Abeta | GBA1 PD | 10 | 1.300 | 0.397 | Inf | 3.270 | 0.001 |
| Normal Abeta - Low Abeta | LRRK2 PD | 10 | 1.300 | 0.397 | Inf | 3.270 | 0.001 |

Table S15: Sample Sizes by Diagnosis and dichotomized Aβ Status

|  | | |
| --- | --- | --- |
| **Diagnosis** | **Normal /High Aβ (n)** | **Low Aβ (PD+) (n)** |
| **Healthy Control** | 47 | 95 |
| **Sporadic PD** | 100 | 235 |
| **GBA1 PD** | 11 | 46 |
| **LRRK2 PD** | 40 | 87 |

###

### Table S16: Unstandardized & Standardized regression coefficients (β) for the fixed effects on cognitive decline (MoCA) over time in the CSF AD Biomarker model

|  | Unstandardized partial regression coefficients | | | Standardized partial regression coefficients | | |
| --- | --- | --- | --- | --- | --- | --- |
| Factors | **Estimates** | **95% CI** | **p** | **Estimates** | **95% CI** | **p** |
| (Intercept) | 30.09 | 28.32 – 31.86 | **<0.001** | 27.41 | 26.42 – 28.39 | **<0.001** |
| Diagnosis [Sporadic] | -0.77 | -1.86 – 0.32 | 0.16 | -0.79 | -1.88 – 0.30 | 0.16 |
| Diagnosis [*GBA1*] | -0.65 | -2.32 – 1.02 | 0.45 | -0.66 | -2.33 – 1.01 | 0.44 |
| Diagnosis [*LRRK2*] | -0.45 | -1.93 – 1.04 | 0.55 | -0.45 | -1.93 – 1.04 | 0.56 |
| Time | 0.24 | -0.10 – 0.58 | 0.16 | -0.30 | -0.47 – -0.12 | **0.001** |
| pTau at Baseline | -0.01 | -0.06 – 0.03 | 0.56 | -0.01 | -0.06 – 0.03 | 0.56 |
| Aβ42 at Baseline | 0.00 | 0.00 – 0.00 | **0.03** | 0.00 | 0.00 – 0.00 | **0.03** |
| Age at Baseline | -0.07 | -0.09 – -0.05 | **<0.001** | -0.61 | -0.78 – -0.43 | **<0.001** |
| Years of Education | 0.11 | 0.06 – 0.16 | **<0.001** | 0.35 | 0.18 – 0.51 | **<0.001** |
| Sex (Male) | -0.44 | -0.79 – -0.10 | **0.01** | -0.44 | -0.79 – -0.10 | **0.01** |
| Duration of PD at Baseline | -0.07 | -0.15 – 0.01 | 0.07 | -0.20 | -0.41 – 0.02 | 0.08 |
| Diagnosis [Sporadic] × Time | 0.36 | 0.16 – 0.56 | **<0.001** | 0.36 | 0.16 – 0.56 | **<0.001** |
| Diagnosis [*GBA1*] × Time | -0.16 | -0.53 – 0.21 | 0.40 | -0.17 | -0.53 – 0.20 | 0.37 |
| Diagnosis [*LRRK2*] × Time | 0.17 | -0.17 – 0.51 | 0.34 | 0.17 | -0.17 – 0.51 | 0.33 |
| Diagnosis [Sporadic] × pTau at Baseline | 0.00 | -0.06 – 0.07 | 0.90 | 0.01 | -0.06 – 0.07 | 0.87 |
| Diagnosis [*GBA1*] × pTau at Baseline | -0.02 | -0.12 – 0.09 | 0.76 | -0.02 | -0.12 – 0.09 | 0.78 |
| Diagnosis [*LRRK2*] × pTau at Baseline | -0.04 | -0.14 – 0.05 | 0.39 | -0.04 | -0.14 – 0.05 | 0.39 |
| pTau at Baseline × Time | -0.00 | -0.01 – 0.00 | 0.49 | -0.00 | -0.01 – 0.00 | 0.48 |
| Aβ42 at Baseline × Time | 0.00 | 0.00 – 0.00 | **<0.001** | 0.00 | 0.00 – 0.00 | **<0.001** |
| Age at Baseline × Time | -0.01 | -0.02 – -0.01 | **<0.001** | -0.11 | -0.14 – -0.08 | **<0.001** |
| Years of Education × Time | 0.01 | 0.00 – 0.02 | **<0.01** | 0.04 | 0.01 – 0.07 | **0.010** |
| Duration of PD at Baseline × Time | 0.02 | 0.01 – 0.04 | **<0.01** | 0.06 | 0.02 – 0.10 | **<0.01** |
| (Diagnosis [Sporadic] × Time) × pTau at Baseline | -0.04 | -0.05 – -0.02 | **<0.001** | -0.04 | -0.05 – -0.02 | **<0.001** |
| (Diagnosis [*GBA1*] × Time) × pTau at Baseline | -0.01 | -0.04 – 0.01 | 0.28 | -0.01 | -0.04 – 0.01 | 0.30 |
| (Diagnosis [*LRRK2*] × Time) × pTau at Baseline | -0.02 | -0.04 – 0.00 | 0.12 | -0.02 | -0.04 – 0.00 | 0.12 |

Abbreviations: *APOE*, Apolipoprotein E; *GBA1*, Glucocerebrosidase mutation; *LRRK2*, Leucine-rich repeat kinase 2

Table S17: Type III ANOVA Results for Mixed Effects dichotomized CSF pTau Biomarker Model

|  | **Sum Sq** | **Mean Sq** | **NumDF** | **DenDF** | **F value** | **Pr(>F)** |
| --- | --- | --- | --- | --- | --- | --- |
| **Diagnosis** | 78.8322 | 26.2774 | 3 | 682.0175 | 9.6111 | 0.0000 |
| **Time** | 46.7196 | 46.7196 | 1 | 360.5513 | 17.0880 | 0.0000 |
| **pTau Group** | 0.5988 | 0.5988 | 1 | 656.7046 | 0.2190 | 0.6399 |
| **Age at Baseline** | 154.0809 | 154.0809 | 1 | 653.0675 | 56.3561 | 0.0000 |
| **Years of Education** | 65.6265 | 65.6265 | 1 | 666.4218 | 24.0033 | 0.0000 |
| **Sex** | 13.0320 | 13.0320 | 1 | 654.2789 | 4.7665 | 0.0294 |
| **Duration of PD** | 0.0239 | 0.0239 | 1 | 638.3101 | 0.0088 | 0.9255 |
| **Diagnosis x Time** | 83.5088 | 27.8363 | 3 | 439.5309 | 10.1813 | 0.0000 |
| **Timex pTau Group** | 15.5577 | 15.5577 | 1 | 365.8972 | 5.6903 | 0.0176 |
| **Time x Age at Baseline** | 178.5472 | 178.5472 | 1 | 355.3815 | 65.3048 | 0.0000 |
| **Timex Years of Education** | 11.2739 | 11.2739 | 1 | 385.4664 | 4.1235 | 0.0430 |
| **Timex Duration of PD** | 13.0455 | 13.0455 | 1 | 308.9696 | 4.7715 | 0.0297 |

Table S18: Difference in annual rate of MoCA decline associated with baseline Aβ42 across PD subgroups

| **PD subgroup** | **Contrast** | **Difference in annual**  **MoCA decline (points/year)** | **95% CI** | **p value** |
| --- | --- | --- | --- | --- |
| Sporadic PD | Low Aβ42 − Mean Aβ42 | -0.1 | -0.14 to -0.07 | **<0.001** |
| ***GBA1*** PD | Low Aβ42 − Mean Aβ42 | -0.1 | -0.14 to -0.07 | **<0.001** |
| ***LRRK2*** PD | Low Aβ42 − Mean Aβ42 | -0.1 | -0.14 to -0.07 | **<0.001** |

Table S19: Difference in annual rate of MoCA decline associated with baseline pTau across PD subgroups

| **PD subgroup** | **Contrast** | **Difference in annual**  **MoCA decline (points/year)** | **95% CI** | **p value** |
| --- | --- | --- | --- | --- |
| Sporadic PD | High pTau − Mean pTau | -0.24 | -0.31 to -0.18 | **<0.001** |
| ***GBA1*** PD | High pTau − Mean pTau | -0.10 | -0.26 to 0.05 | 0.18 |
| ***LRRK2*** PD | High pTau − Mean pTau | -0.13 | -0.26 to 0.01 | **0.06** |

### Table S20: Distribution of MoCA Assessment Visits Across PD Subgroups

| **Number of Visits** | ***GBA1* PD (%)** | ***LRRK2* PD (%)** | **Sporadic PD (%)** |
| --- | --- | --- | --- |
| 2 Visits | 14.8 | 7.9 | 15.5 |
| 3 Visits | 19.7 | 8.5 | 30.5 |
| 4 Visits | 11.4 | 12.7 | 17.2 |
| 5 Visits | 10.7 | 11.5 | 12.4 |
| 6 Visits | 18.9 | 15.2 | 11.8 |
| 7 Visits | 13.9 | 9.7 | 1.7 |
| 8 Visits | 2.5 | 9.7 | 2.1 |
| 9 Visits | 3.3 | 12.1 | 2.6 |
| 10 Visits | 0.8 | 6.1 | 1.6 |
| 11 Visits | 0.8 | 1.8 | 2.3 |

Abbreviations: *GBA1*, Glucocerebrosidase mutation; *LRRK2*, Leucine-rich repeat kinase 2

**Supplementary Figures**

Figure S1: Effect of baseline age (Mean ± SD) on MoCA scores across PD subgroups and *APOE* genotypes over time


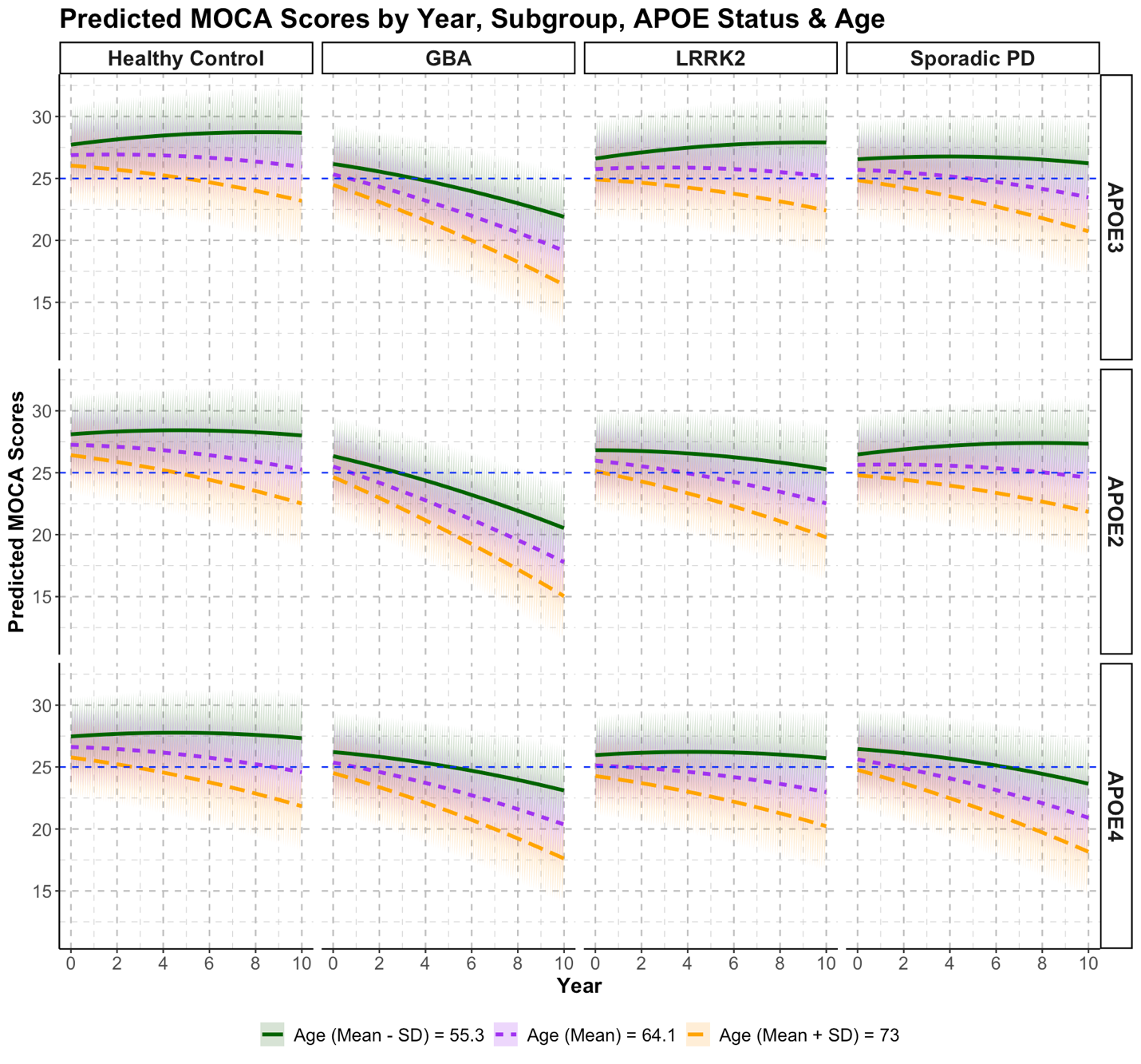


### The figure illustrates the values of MOCA predicted by model fixed effects over time across PD subgroups, Healthy controls and *APOE* genotypes, at the grand mean of age (64.1 years, dotted purple line) and for ages 1 SD above (73 years, dashed orange line) and below (55.3 years, solid green line) the mean. These predictions highlight the impact of age on the trajectory of cognitive decline, with older individuals experiencing a steeper decline. All other fixed effect variables are held constant at their mean values. Colored shading indicates 95% confidence intervals.

Figure S2: Predicted MoCA score trajectories in *APOE* ε4 carriers vs ε4 non-carriers across PD subgroups and Healthy Controls


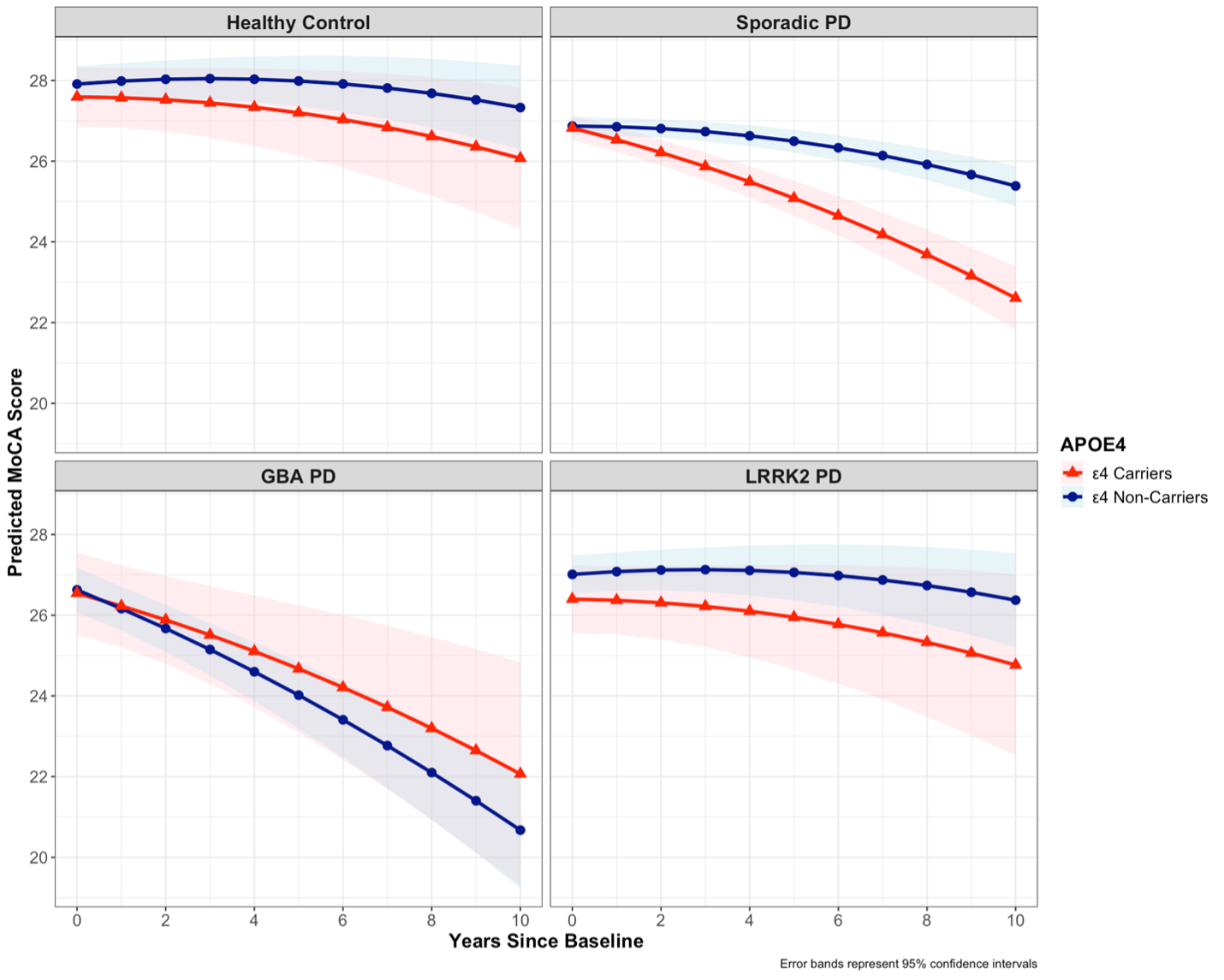


### Values of MoCA scores across years in the study predicted by model fixed effects holding constant covariates of baseline age, sex, education and duration of PD at baseline. Data shown reflect results for mean age (64.2 years), male gender, education >12 years and mean PD duration at baseline (1.68 years) in *APOE* ε4 carriers vs ε4 non- carriers across PD subgroups. Colored bands indicate 95% confidence intervals.

Figure S3: Predicted MoCA score trajectories in *APOE* ε4 carriers vs ε4 non-carriers across PD subgroups and Healthy Controls with cohort level included as a fixed effect in the model


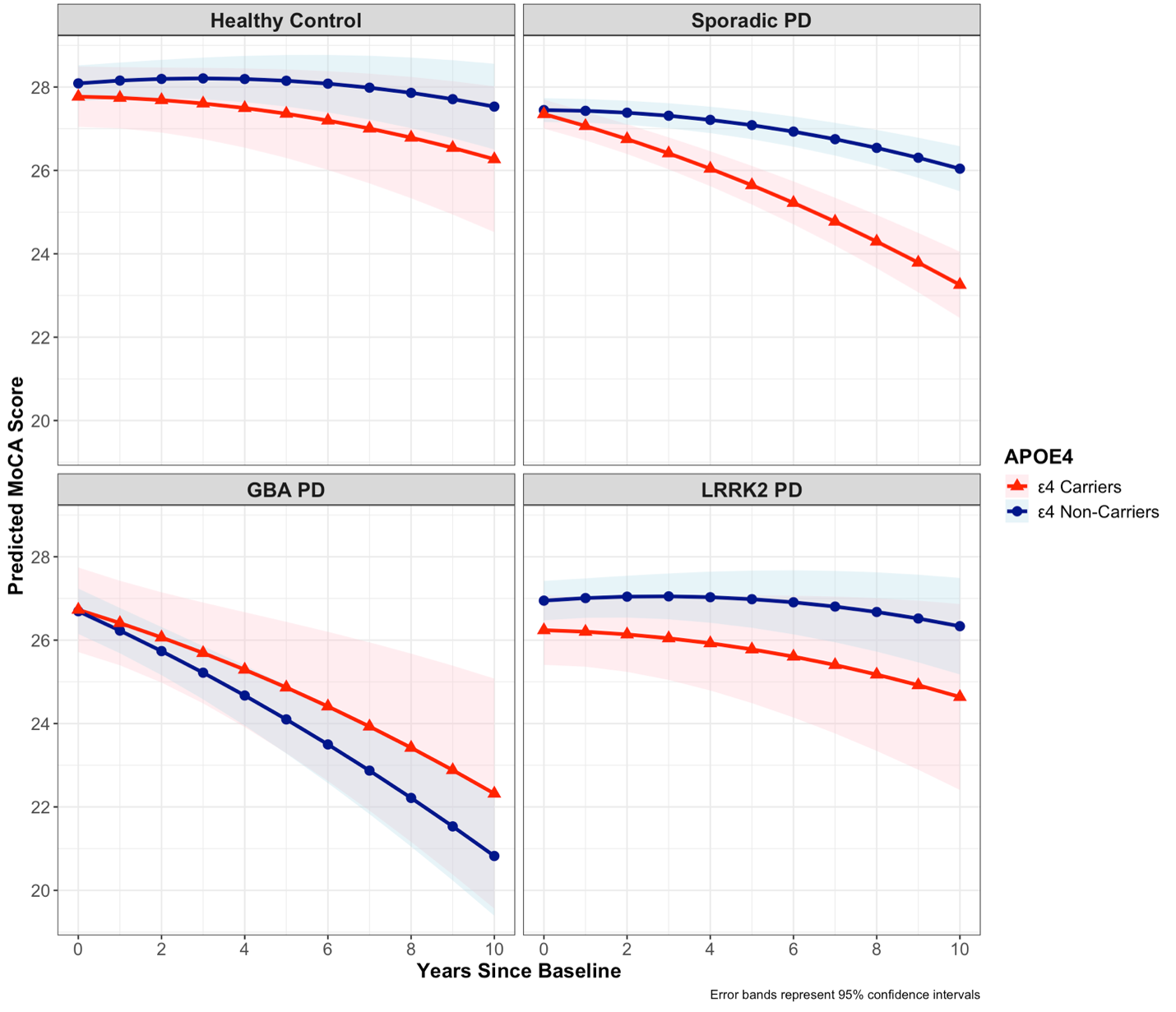


### Values of MoCA scores across years in the study predicted by model fixed effects, holding covariates baseline age, sex, cohort level and education constant. Data shown reflect results for mean age (64.2 years), male gender, and education >12 years in *APOE* ε4 carriers vs. ε4 non-carriers across PD subgroups in the PPMI cohort. Colored bands indicate 95% confidence intervals.

Figure S4: Predicted MoCA trajectories by dichotomized baseline CSF amyloid-β status across diagnostic groups

**
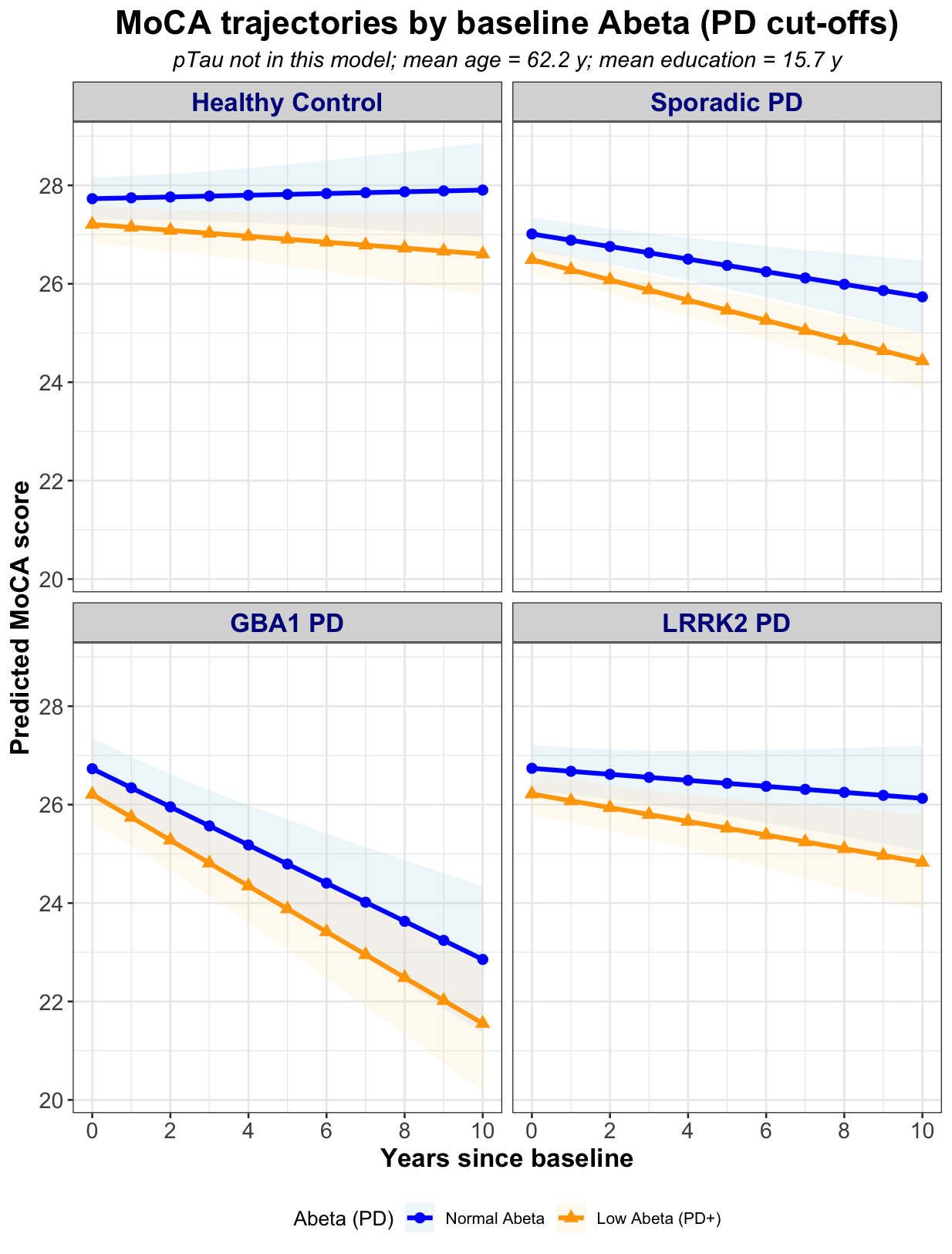
**

### Model-predicted MoCA scores adjusted for baseline age, sex, education. Amyloid-β status was defined using PD-specific cut-offs. Across all diagnostic groups, participants in the low Aβ42 group exhibited a significantly steeper rate of cognitive decline, compared with those in the normal Aβ42 group. Values represent fixed-effect predictions with 95% confidence intervals where age set at mean age =62.2 years, male gender and mean years of education = 15.7 years

Figure S5: Predicted MoCA trajectories by dichotomized baseline CSF ptau status across diagnostic groups

**
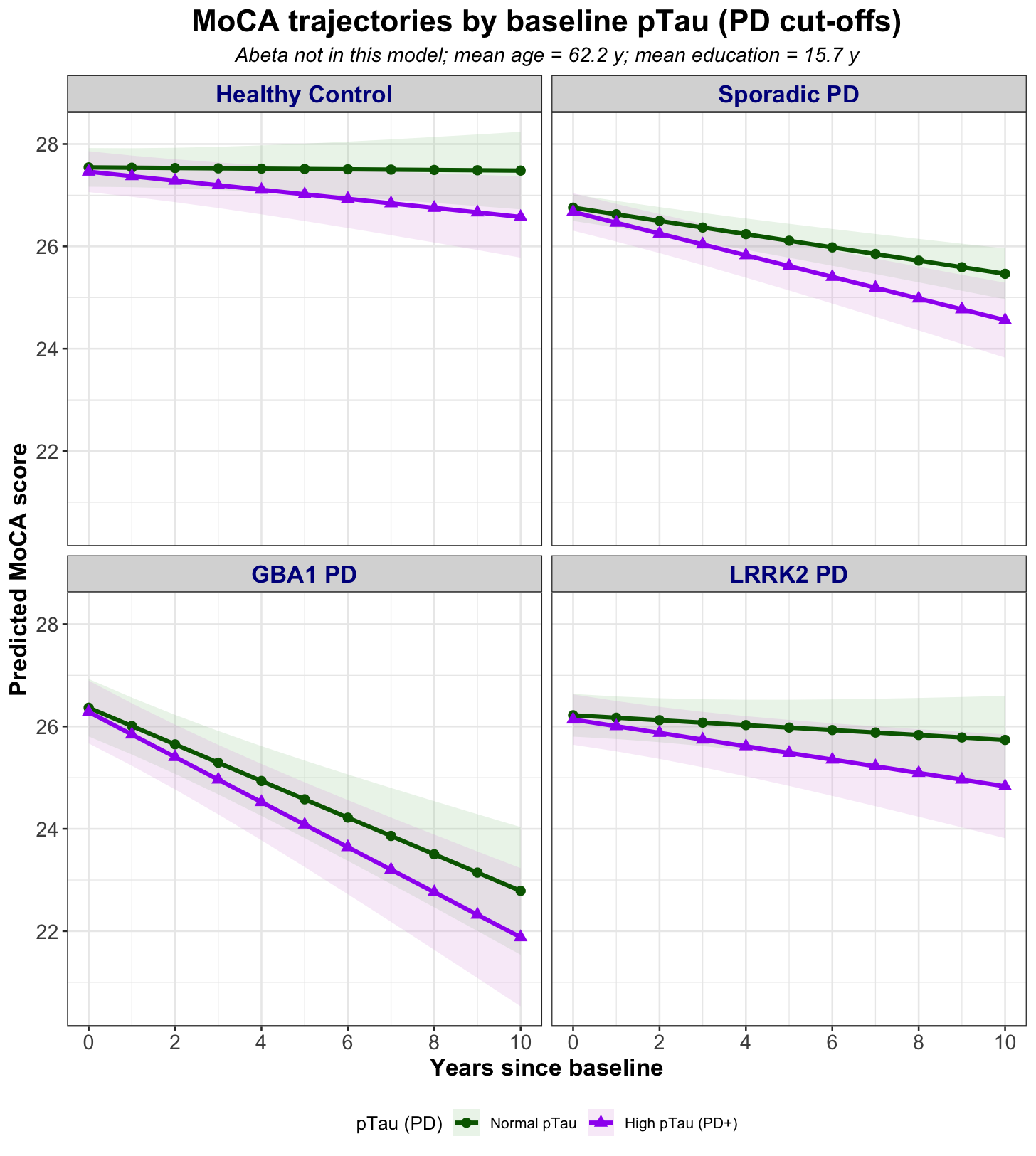
**

### Model-predicted MoCA scores adjusted for baseline age, sex, education. pTau was defined using PD-specific cut-offs. In all diagnostic groups, individuals in the high pTau group demonstrated significantly steeper rate of cognitive decline than those in the low pTau group (Age set at mean age = 62.2 years, male gender and mean years of education =15.7 years) Values represent fixed-effect predictions with 95% confidence intervals.

Figure S6: Raw Data Spaghetti Plots of MoCA Scores by Diagnosis Group


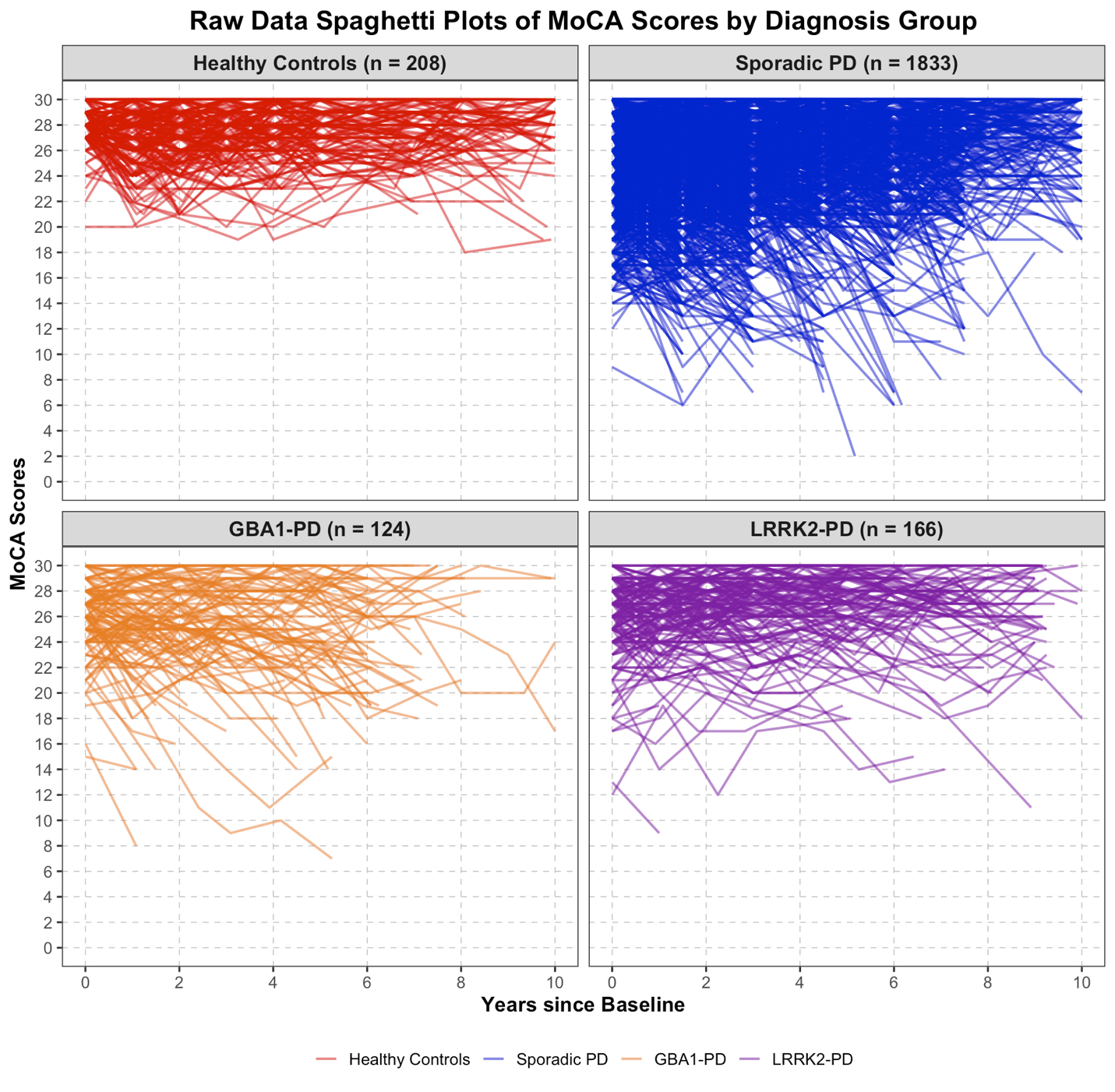


Each line represents the trajectory of MOCA score over time for a single participant.
